# Spontaneous, persistent, T cell–dependent IFN-γ release in patients who progress to Long Covid

**DOI:** 10.1126/sciadv.adi9379

**Published:** 2024-02-21

**Authors:** Benjamin A. Krishna, Eleanor Y. Lim, Marina Metaxaki, Sarah Jackson, Lenette Mactavous, NIHR BioResource, Paul A. Lyons, Rainer Doffinger, John R. Bradley, Kenneth G. C. Smith, John Sinclair, Nicholas J. Matheson, Paul J. Lehner, Nyaradzai Sithole, Mark R. Wills

**Affiliations:** ^1^ Cambridge Institute of Therapeutic Immunology and Infectious Disease (CITIID), Cambridge CB2 0AW, UK.; ^2^ Department of Medicine, University of Cambridge, Cambridge CB2 0QQ, UK.; ^3^ Department of Infectious Diseases, Cambridge University Hospitals NHS Foundation Trust, Cambridge CB2 0QQ, UK.; ^4^ NIHR BioResource, Cambridge University Hospitals, Cambridge Biomedical Campus, Cambridge CB2 0QQ, UK.; ^5^ Department of Clinical Biochemistry and Immunology, Cambridge University Hospitals NHS Foundation Trust, Cambridge CB2 0QQ, UK.; ^6^ National Institute for Health Research (NIHR) Cambridge Biomedical Research Centre, Cambridge CB2 0QQ, UK.; ^7^ Department of Renal Medicine, Cambridge University Hospitals NHS Foundation Trust, Cambridge CB2 0QQ, UK.; ^8^ NHS Blood and Transplant, Cambridge CB2 0PT, UK.

## Abstract

After acute infection with severe acute respiratory syndrome coronavirus-2 (SARS-CoV-2), a proportion of patients experience persistent symptoms beyond 12 weeks, termed Long Covid. Understanding the mechanisms that cause this debilitating disease and identifying biomarkers for diagnostic, therapeutic, and monitoring purposes are urgently required. We detected persistently high levels of interferon-γ (IFN-γ) from peripheral blood mononuclear cells of patients with Long Covid using highly sensitive FluoroSpot assays. This IFN-γ release was seen in the absence of ex vivo peptide stimulation and remains persistently elevated in patients with Long Covid, unlike the resolution seen in patients recovering from acute SARS-CoV-2 infection. The IFN-γ release was CD8^+^ T cell–mediated and dependent on antigen presentation by CD14^+^ cells. Longitudinal follow-up of our study cohort showed that symptom improvement and resolution correlated with a decrease in IFN-γ production to baseline levels. Our study highlights a potential mechanism underlying Long Covid, enabling the search for biomarkers and therapeutics in patients with Long Covid.

## Teaser

Patients with Long Covid symptoms produce an antiviral protein called interferon-γ persistently until their symptoms resolve.

## INTRODUCTION

A substantial proportion of people infected with severe acute respiratory syndrome coronavirus-2 (SARS-CoV-2) exhibit persistent, distressing symptoms and/or the emergence of new symptoms after COVID-19. These are difficult to delineate into specific endotypes and are broadly grouped as post-COVID syndrome, post-acute sequelae of SARS-CoV-2 (PASC), or Long Covid. There is a lack of consensus for diagnosing Long Covid, and causes of Long Covid remain unclear, with resultant lack of approved pharmacological therapeutic interventions for this debilitating condition. Long Covid has a plethora of relapsing and remitting symptoms (*1*) and multisystemic organ involvement (*2*–*4*) with prevalence rates ranging from 0.2 to 33% (*5*–*8*). It is likely that some differences between studies are due to differences in diagnostic criteria and definition of Long Covid (*9*). As the pathogenesis of this heterogeneous disease is unclear, there is also a lack of diagnostic biomarkers and treatments. Adding to the diagnostic conundrum is the mounting evidence that Long Covid does not correlate with severity of the preceding acute illness, as it can affect patients who were asymptomatic and/or had mild SARS-CoV-2 infections (*10*). Although some patients improve without therapeutic interventions, a large proportion of patients have not improved and/or have relapsing and worsening symptoms. Anecdotal reports from some patients with Long Covid suggest that vaccination either improved or ameliorated their symptoms; however, the pathophysiological basis for these findings remains unknown (*11*, *12*).

In view of the urgent clinical need to determine the pathophysiological basis of Long Covid and ascertain the relevant biomarker/s to aid diagnosis and assist with objective disease monitoring, we studied cytokine secretion from peripheral blood mononuclear cells (PBMCs). These PBMCs were either from a cohort of undifferentiated patients diagnosed with Long Covid (as defined by clinical symptoms), controls of never-infected individuals, or from a longitudinal follow-up of those acutely infected who did not develop Long Covid. In addition, to better understand the disease process and immune parameters, we performed immunophenotyping combined with intracellular cytokine staining of donor PBMCs. We hereby show that SARS-CoV-2 infection induces a large increase in cluster of differentiation 8–positive (CD8^+^) T cell–mediated interferon-γ (IFN-γ) release, as well as smaller increases in other proinflammatory cytokines, not requiring any ex vivo peptide stimulation. This state persists for several months after acute SARS-CoV-2 infection but then returns to baseline in healthy individuals. By contrast, spontaneous release of IFN-γ from PBMCs of patients with Long Covid remains high. Long Covid symptom recovery is associated with IFN-γ levels returning to baseline, indicating a correlation between IFN-γ levels and Long Covid symptoms. We also determine that this IFN-γ production by CD8^+^ T cells requires antigen presentation by CD14^+^ monocytes and major histocompatibility complex (MHC) class I contact. Together, we identify IFN-γ release as a potential biomarker in some patients with Long Covid and highlight an immunological mechanism underlying this debilitating disease, which will potentially pave the way for the development of much needed therapies.

## RESULTS

### SARS-CoV-2 infection induces spontaneous and persistent IFN-γ production in a subset of patients with Long Covid

**Fig. 1.**
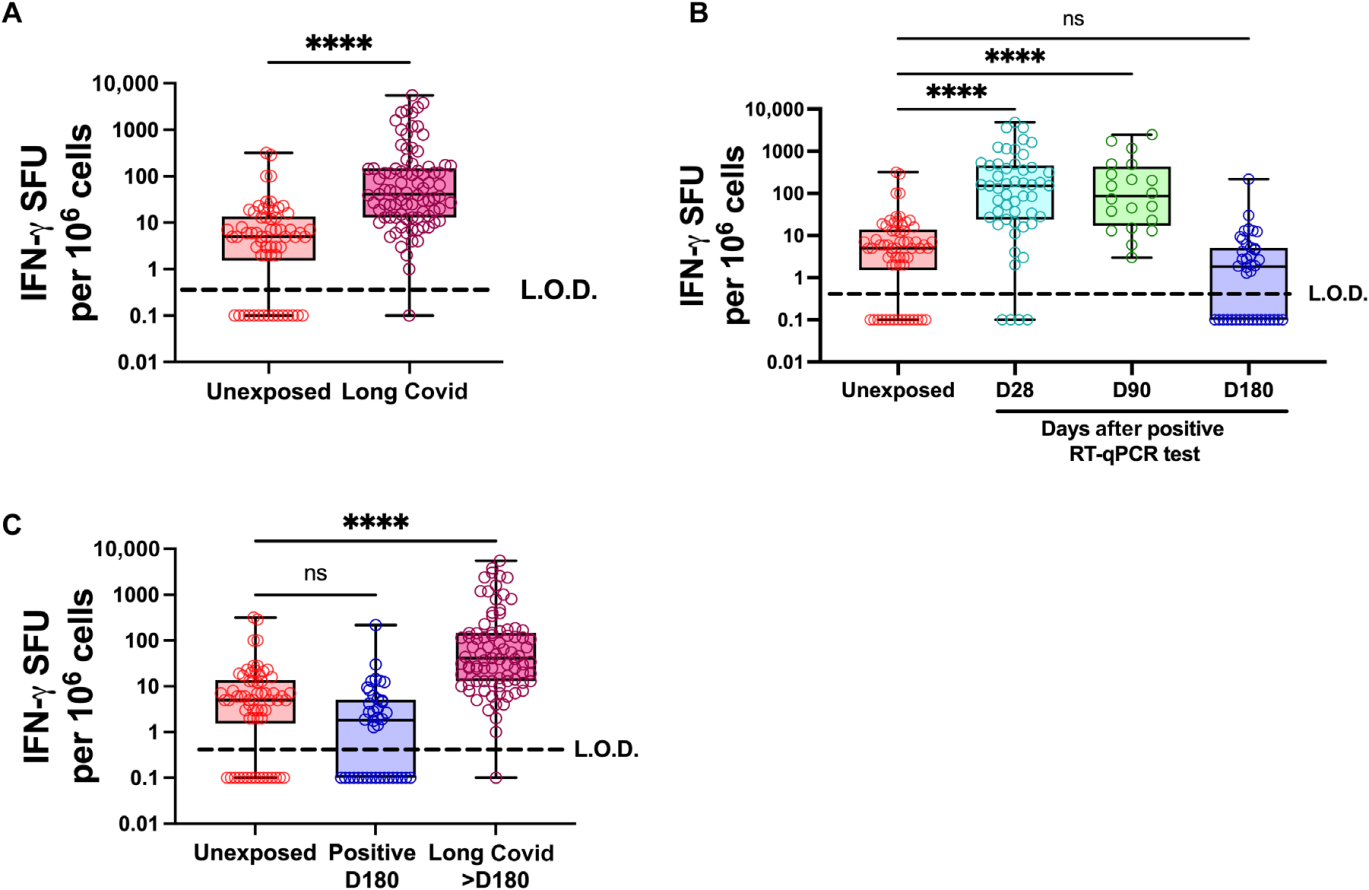
Spontaneous IFN-γ release is triggered by SARS-CoV-2 infection and fails to resolve to baseline in patients with diagnosed Long Covid. (**A** to **C**) PBMCs were isolated from the blood of negative control unexposed donors (red, *n* = 54), patients diagnosed with Long Covid (burgundy, *n* = 87), and positive-control RT-qPCR–confirmed donors at 28, 90, or 180 days after PCR test (cyan, *n* = 51; green, *n* = 20; blue, *n* = 40). These PBMCs were not stimulated with any peptides ex vivo. After 48 hours of incubation, IFN-γ release was measured by FluoroSpot assay as spot forming units (SFU) per million PBMCs. Each point represents the mean from a single donor, where donor samples were run in duplicate, and zero results were set as 0.1 to allow their inclusion on a log scale. L.O.D., limit of detection. We compared unexposed and Long Covid cohorts (A), unexposed against each time point for the cohort who experienced acute COVID-19 (B), and then unexposed, acute COVID-19 at day 180 and the Long Covid cohort specifically (C). Significance calculated by two-way Kruskal-Wallis analysis of variance (ANOVA), with Dunn’s multiple comparisons test, ****P < 0.0001. ns, not significant.

We recently reported a highly sensitive T cell FluoroSpot assay that measured SARS-CoV-2 specific interleukin-2 (IL-2) and IFN-γ responses in patients with Long Covid (*13*). The release of cytokines following stimulation of patient-derived PBMC with SARS-CoV-2 antigens allowed us to determine the frequency of T cells specific for overlapping peptide pools of spike (S), nucleocapsid (N), and membrane (M) proteins in patients with Long Covid (*13*). In addition to using in vitro peptides to stimulate cytokine release from PBMC, we also measured spontaneous cytokine release in the absence of any stimulation with SARS-CoV-2 antigens. The detection of cytokine-secreting cells from unstimulated PBMC is usually very low (*14*), but a significantly higher proportion/percentage of cells producing IFN-γ was clearly observed in PBMCs from patients with Long Covid as compared to unexposed prepandemic, historic negative control samples taken between 2014 and 2019 (Fig. 1A and fig. S1).

We thus compared PBMC samples from patients with Long Covid who had been symptomatic for at least 6 months (median symptom duration of 7 months) to PBMC samples from prepandemic negative controls (Fig. 1A). As an additional control for acute SARS-CoV-2 infection, we also examined PBMC from patients taken at 28, 90, and 180 days after positive SARS-CoV-2 reverse transcription quantitative polymerase chain reaction (RT-qPCR). PBMC from SARS-CoV-2–infected individuals showed a substantially higher frequency of spontaneous IFN-γ–positive cells at 28 and 90 days after positive PCR as compared to uninfected controls. By 180 days, this IFN-γ release had resolved, with IFN-γ–producing cells at a similar level as uninfected controls (Fig. 1B). However, a high frequency of IFN-γ release persisted in patients with Long Covid even beyond 180 days of symptom onset (Fig. 1C). This phenotype was also specific for IFN-γ, as we saw no change in IL-2 production on the same FluoroSpot plates (fig. S2).

To understand the functionality of T cells in patients with Long Covid, we also treated PBMCs from each cohort with a peptide pool covering T cell epitopes from human cytomegalovirus, Epstein-Barr virus, and influenza (CEF) (fig. S3, A and B). We also wanted to confirm that T cells from every patient were able to make IL-2 and IFN-γ responses and to do this treated PBMCs with anti-CD3 antibody, *Staphylococcus* Enterotoxin B (SEB), phytohemagglutinin (PHA), and lipopolysaccharide (LPS) (fig. S3, C and D). Overall, there were no significant differences between the cohorts, but the high spontaneous IFN-γ production in day 28, day 90, and Long Covid samples affected the ability to measure IFN-γ production to CEF above background levels in these groups (fig. S3B). Overall, these data suggest that the patients with Long Covid still had functioning T cells.

As we noticed that spontaneous IFN-γ production began during acute infection, we wanted to understand whether IFN-γ production correlated with disease severity. To do this, we stratified our donors at day 28 into severity categories originally established by Bergamaschi *et al.* (*15*). We saw that spontaneous IFN-γ production did not correlate with disease severity for acute illness (fig. S4).

### IFN-γ production is maintained by individuals who do not recover from Long Covid

**Fig. 2.**
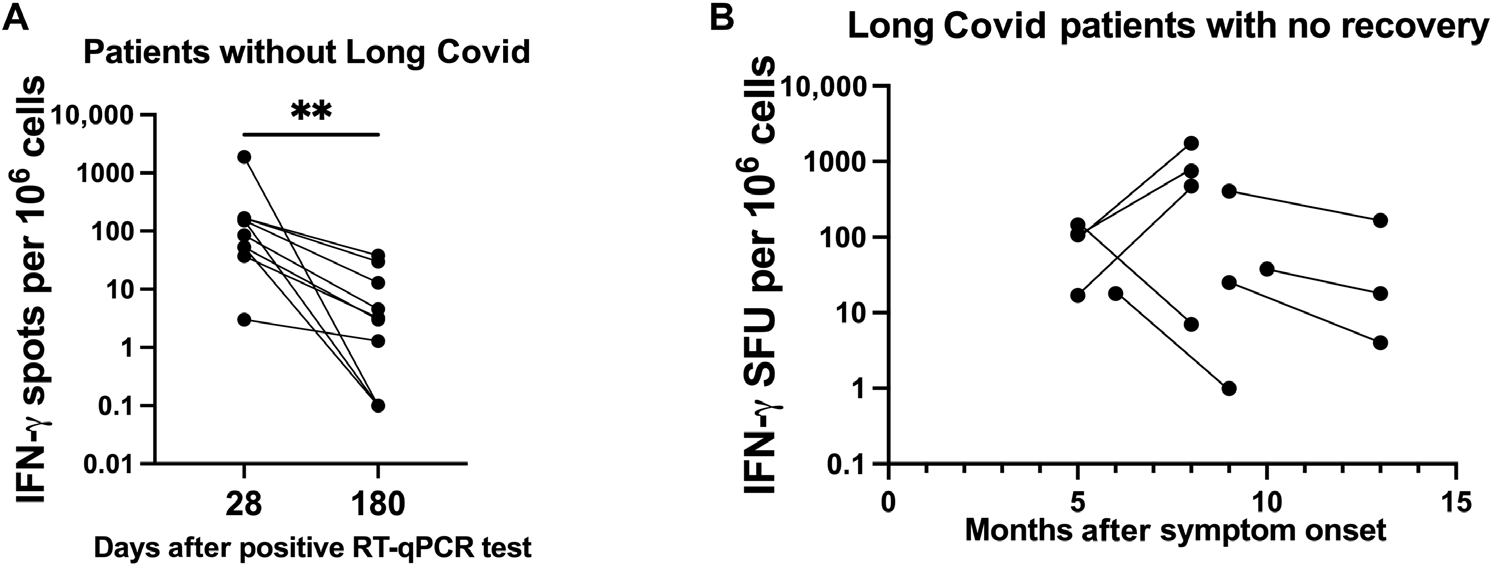
Spontaneous IFN-γ production resolves in individuals with symptom resolution. (**A**) Data from Fig. 1B were replotted for PBMCs from 10 donors who gave samples at 28 and 180 days after positive RT-qPCR result and reported symptom recovery. (**B**) IFN-γ release was measured by FluoroSpot assay for patients with Long Covid who did not report symptom resolution. Results are plotted at time after symptom onset. (A) and (B) IFN-γ release was quantified as spot forming units per million PBMCs. Each point represents the mean from a single donor, where donor samples were run in duplicate and zero results were set as 0.1 to allow their inclusion on a log scale. Significance calculated by or two-way Wilcoxon signed-rank test for (A) where **P < 0.01.

Our analysis in Fig. 1 used longitudinal cohorts of different patients with only some overlap at each time point. We therefore tracked changes in unstimulated IFN-γ release across individual patients over time. Unstimulated IFN-γ release in PBMCs from the same 10 patients with no Long Covid symptoms showed a clear decrease in spontaneous IFN-γ release by day 180 (Fig. 2A). By contrast, when we recalled some patients with Long Covid who had not recovered for a second blood donation, unstimulated IFN-γ release across these 16 patients was persistent, with no overall downward trend but large patient to patient variation (Fig. 2B). Together, these data show that unstimulated IFN-γ release usually resolves following acute infection but persists in the cohort of patients who progress to Long Covid.

### SARS-CoV-2–induced IFN-γ is produced by activated CD8^+^ T cells requiring contact with CD14^+^ cells

**Fig. 3.**
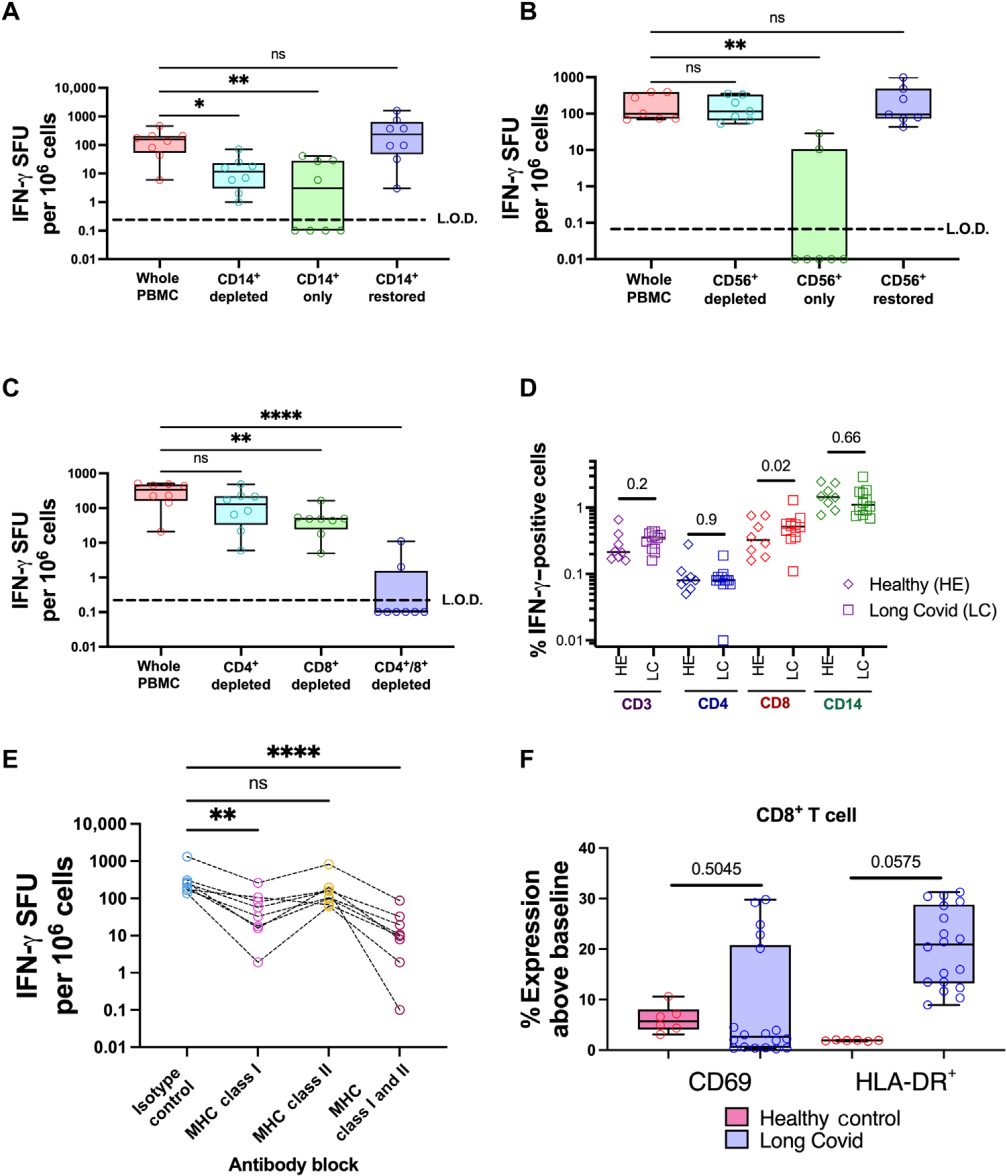
Spontaneous IFN-γ release from the PBMCs of patients with Long Covid is caused by CD8^+^ T cell interaction with CD14^+^ cells via MHC class I peptide presentation. (**A** to **C**) MACS separation was used to isolate CD14^+^, CD56^+^, CD4^+^, or CD8^+^ cells from PBMC donations from patients with Long Covid. Whole PBMCs (red) or various combinations of depleted PBMCs, isolated cell populations, or depleted PBMCs with the isolated populations added back in from eight patients diagnosed with Long Covid were plated without peptide stimulation for 48 hours to measure IFN-γ release by FluoroSpot assay. (**D**) PBMCs from patients diagnosed with Long Covid (LC; *n* = 12) or healthy controls (HE; *n* = 8) were incubated for 24 hours with brefeldin A and monensin to allow intracellular accumulation of IFN-γ. Cells were then stained for CD3, CD4, CD8, CD14, and IFN-γ to compare IFN-γ production in these cells types between healthy controls and Long Covid. (**E**) CD14^+^ cells from eight donors were isolated by MACS separation and incubated for 1 hour with anti–MHC class I, anti–MHC class II, or isotype control antibodies. The cells were then washed and returned to the donor PBMCs, and IFN-γ release was measured by FluoroSpot assay after 24 hours of incubation. (A to C) IFN-γ release was quantified as in Figs. 1 and 2. Significance calculated by Kruskal-Wallis two-way ANOVA [(A) to (C) and (E)] or Friedman two-way ANOVA test (D) with Dunn’s multiple comparison test, *P < 0.05, **P < 0.01, and ****P < 0.0001. (D) Data are shown as percentage of cells that were positive for IFN-γ. Significance was calculated using multiple two-way Mann-Whitney *U* tests. (**F**) Activation markers, CD69 and Human Leukocyte Antigen–DR isotype (HLA-DR), on CD8^+^ T cells were measured in 18 patients with Long Covid and 8 healthy donors using the gating strategy shown in fig. S7. Significance, was measured by Kruskal-Wallis ANOVA.

To determine the cell types that produce IFN-γ in patients with Long Covid, we conducted a series of independent cell depletion assays. We removed CD4^+^, CD8^+^, CD14^+^, or CD56^+^ cells from donor PBMCs by magnetic-activated cell sorting (MACS) and measured IFN-γ release from these cell populations by FluoroSpot assay. Depletion of CD14^+^ cells reduced IFN-γ release in all cases. However, the isolated CD14^+^ cells from the same donor release negligible IFN-γ, suggesting that they are required for IFN-γ production but are not the source of IFN-γ (Fig. 3A). The addition of isolated CD14^+^ cells back into the CD14^+^ cell–depleted PBMC population restored IFN-γ production (Fig. 3A). Removal of CD56^+^ cells [predominantly natural killer (NK) and NKT cells] had no significant effect on IFN-γ production, suggesting that these cell types are unlikely to be involved (Fig. 3B).

CD8^+^ T cells and subpopulations of CD4^+^ T cells are a major source of IFN-γ. We performed the same cell depletion assays on these populations and found that depletion of CD8^+^ cells significantly decreased IFN-γ production. A much smaller decrease was noted after CD4^+^ cell depletion, which was not statistically significant alone but did appear to be additive after CD8^+^ cell depletion (Fig. 3C). Together, our data suggest that CD8^+^ T cells in contact with CD14^+^ cells are required for unstimulated IFN-γ production and the majority of this is produced by CD8^+^ T cells in PBMCs from patients with Long Covid.

To further validate our findings, we used intracellular flow cytometry to costain for IFN-γ with markers of cellular differentiation status. Comparing patients with Long Covid to healthy controls, we found that IFN-γ was increased in CD3^+^ cells in general and CD8^+^ cells in particular, but not in CD14^+^ or CD4^+^ cells (Fig. 3D). These data validate our FluoroSpot data using subset-depleted PBMC, showing that the IFN-γ release is predominantly CD8^+^ T cell–mediated.

As CD14^+^ cells are required for the release of IFN-γ in patients with Long Covid (Fig. 3A), we therefore tested whether antigen presentation by CD14^+^ cells induced T cell–dependent IFN-γ release. Anti-MHC class I and/or class II antibodies were used to block the cell surface of isolated CD14^+^ cells and subsequently cultured with CD14^+^ cell–depleted autologous PBMC. MHC class I blocking antibodies significantly reduced the frequency of IFN-γ–positive cells, while class II blocking antibodies had only a minor and nonsignificant effect (Fig. 3E). Together, our results suggest that MHC class I–dependent antigen presentation by CD14^+^ cells to CD8^+^ T cells triggers IFN-γ release.

We measured absolute leukocyte subsets using Becton Dickinson Trucount tubes to identify any differences in leukocyte populations between patients with Long Covid and healthy donors. Increased monocyte populations and decreased regulatory T cells were found in patients with Long Covid and a profound decrease in NKG2C^+^ NK cells (fig. S5).

### IFN-γ is the primary cytokine persistently released in patients with Long Covid

**Fig. 4.**
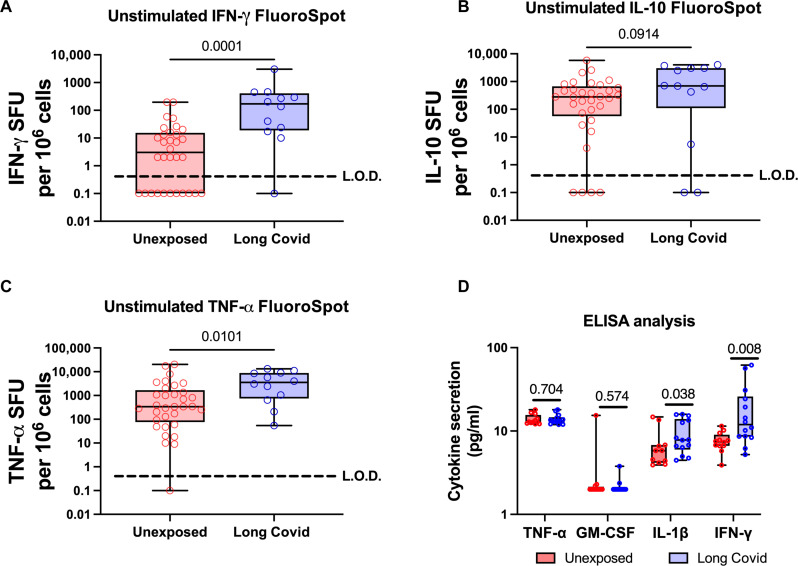
PBMCs from patients with Long Covid produce IFN-γ and TNF-α but not IL-10. PBMCs were isolated from the blood of patients with Long Covid (blue, *n* = 12) and those never infected with SARS-CoV-2 (unexposed, red, *n* = 33). Spontaneous production of IFN-γ (**A**), IL-10 (**B**), and TNF-α (**C**) was measured by FluoroSpot assay on unstimulated cells. After 48 hours of incubation, cytokine release was measured by FluoroSpot assay as spot forming units per million PBMCs. Each point represents the mean from a single donor, where donor samples were run in duplicate and zero results were set as 0.1 to allow their inclusion on a log scale. Significance calculated by two-way Mann-Whitney *U* test. (**D**) We used ELISA assays to confirm increased cytokine production in PBMCs from patients with Long Covid compared to unexposed controls. We tested TNF-α as was seen in (C), as well as GM-CSF, IL-1β, and IFN-γ that were detected by LEGENDplex analysis (fig. S6). Values below the limit of detection (4 pg/ml) were shown as 4 pg/ml to allow their inclusion on a logarithmic scale. Statistical significance was calculated by multiple Mann-Whitney tests, with *P* values showing significance for IL-1β and IFN-γ only.

Given the known generalized cytokine perturbation in SARS-CoV-2 infection, we assessed whether Long Covid specifically correlated with increased IFN-γ or whether PBMCs from patients with Long Covid also released other cytokines. A small increase in tumor necrosis factor–α (TNF-α) but no change in IL-2 or IL-10 (Fig. 4, A to C) was seen using FluoroSpot analysis. A bead-based immunoassay (LEGENDplex, BioLegend) analyzing 25 different cytokines showed a general increase in proinflammatory cytokines (e.g., IL-1β and IL-6), although IFN-γ showed the largest increase in patients with Long Covid (fig. S6). Smaller increases in granulocyte colony-stimulating factor (G-CSF) and granulocyte-macrophage colony-stimulating factor (GM-CSF), which may explain the higher monocyte numbers in fig. S5, were seen. We went on to verify production of these cytokines using enzyme-linked immunosorbent assay (ELISA) analysis. Individual ELISA analysis of TNF-α, IL-1β, GM-CSF, and IFN-γ was able to corroborate increased production of IFN-γ and IL-1β but not TNF-α (which was also not corroborated by LEGENDplex analysis) nor GM-CSF; the production of which was likely below ELISA detection threshold (Fig. 4D).

### Symptom improvement correlates with decreased IFN-γ production

**Fig. 5.**
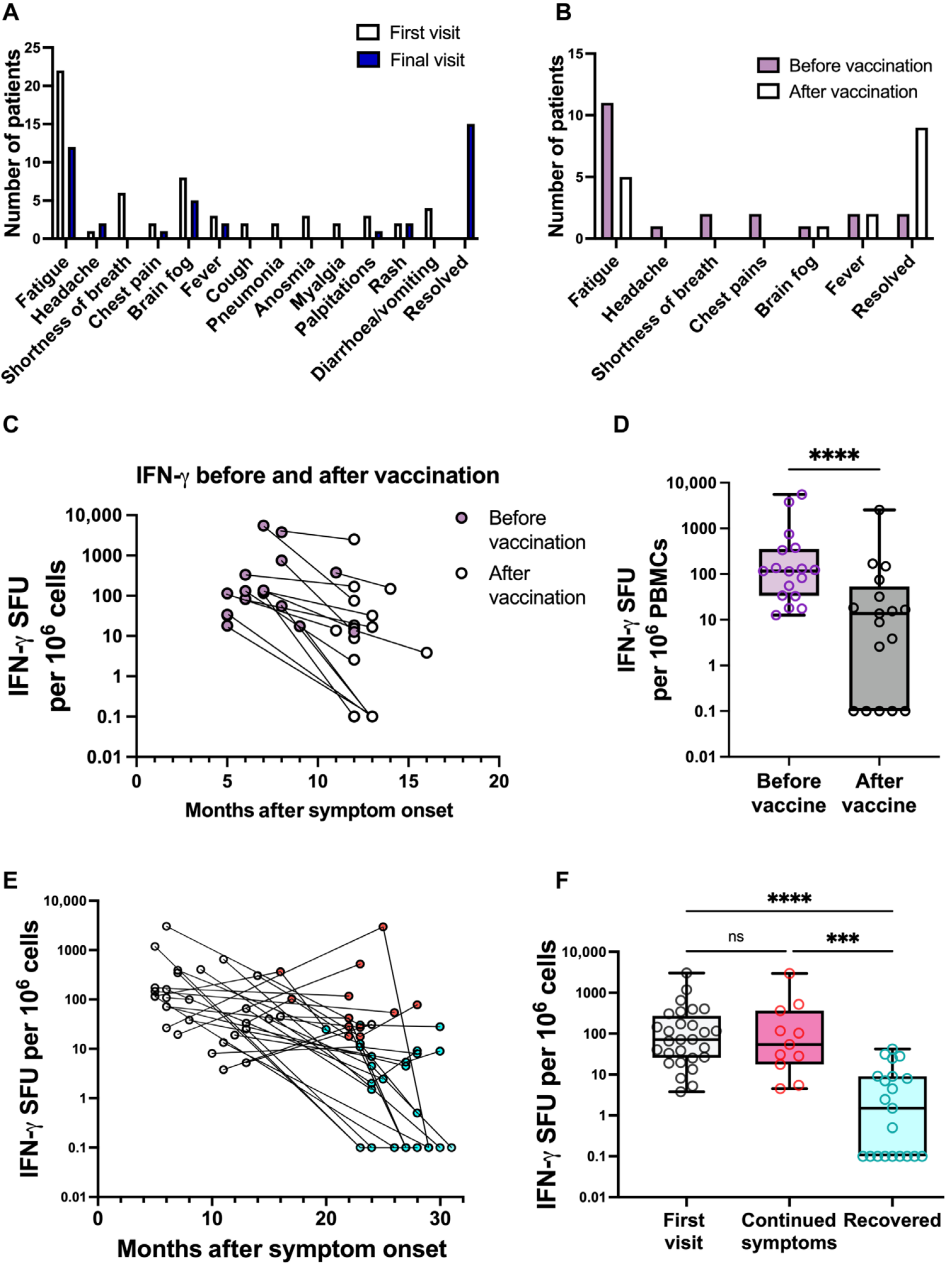
Reduction in Long Covid symptoms correlated with loss of spontaneous IFN-γ release. Longitudinal follow-up revealed that some patients either recovered spontaneously (**A**) or after vaccination (**B**). (**C** to **F**) PBMCs were isolated from patients with Long Covid at the indicated time points after infection, and unstimulated IFN-γ production was measured by FluoroSpot analysis. (C) Seventeen patients reported symptoms improved after vaccination; here, we have plotted IFN-γ release before (pink) and after (gray) vaccination. (D) Unstimulated IFN-γ production reduction was statistically significant after vaccination as calculated by two-way Wilcoxon ranked sign test, ****P < 0.0001. (E) and (F) A further 21 of 34 patients experienced spontaneous symptoms improvement over the course of the study. (E) As in (C), unstimulated IFN-γ production is plotted against time for these patients, where red dots indicate that the patient reported continued symptoms, while cyan dots indicate an improvement or resolution of symptoms. (F) Unstimulated IFN-γ production was significantly lower in patients who recovered from Long Covid symptoms compared to those who had ongoing symptoms, as calculated by Kruskal-Wallis test. ***P < 0.001.

Last, we followed our Long Covid cohort for up to 31 months after acute infection. During this follow-up period, a considerable number of patients experienced resolution of some, if not all, of their symptoms either spontaneously (21 of 34) (Fig. 5A) or after SARS-CoV-2 vaccination (Fig. 5B) (*11*). To investigate whether unstimulated IFN-γ release correlated with some of the symptoms, we measured unstimulated IFN-γ release in patients with Long Covid before and after vaccination and found a significant decrease in IFN-γ after vaccination that correlated with symptom resolution (Fig. 5, C and D). Vaccination response was confirmed by measurement of antispike response (fig. S9). Similarly, we saw a decrease in IFN-γ release across time, with IFN-γ levels significantly reduced in patients reporting an alleviation of their Long Covid symptoms (Fig. 5E) compared to those who reported continued symptoms (Fig. 5F). Together, these data provide evidence that unstimulated IFN-γ release correlates with Long Covid symptoms.

## DISCUSSION

Long Covid, or PASC, is an important, long-term clinical problem that results from COVID-19 infection. With a staggering 600 million COVID-19 infections as of January 2023, the large number of people living with Long Covid will be an enormous burden for health services and will result in meaningfully negative impact on different facets of society as a whole. Understanding the molecular mechanisms underlying Long Covid is therefore of paramount importance, as this will help in designing robust diagnostic and treatment options for patients in need. In this study, we reveal that CD8^+^ T cells from patients diagnosed with Long Covid secrete increased levels of IFN-γ, spontaneously without peptide stimulation, compared to healthy controls. This high IFN-γ secretion correlated with symptom persistence in patients with Long Covid.

Our study shows that initial infection with SARS-CoV-2 triggers increased spontaneous IFN-γ secretion, which resolves in most patients by 6 months after infection. Classification of Long Covid is somewhat controversial with some publications, suggesting that Long Covid begins at 4 weeks after infection, and others begin at 12 weeks or longer. Our data suggest that Long Covid lasting over 6 months could be differentially classified to that lasting 12 weeks based on this IFN-γ signature. Our data suggest that most patients have not resolved their IFN-γ secretion at 3 months after infection, which concurs with other studies that many patients report ongoing symptoms 6 weeks to 6 months after infection (*16*–*18*). It is notable that our cohort of patients with Long Covid was dominated by those experiencing fatigue (Fig. 5, A and B) and tended to have more severe symptoms, having been recruited from a specialist Long Covid clinic. It would be interesting in the future to see whether this IFN-γ signature is seen in patients with more mild symptoms and those with specific long-lasting symptoms, such as anosmia.

We hereby extend the follow-up to 31 months after infection and show that IFN-γ is strongly correlated with Long Covid symptoms, especially the predominant symptom in our cohort: fatigue. We observed that our Long Covid cohort had variable IFN-γ secretion, both within the cohort and in the same patients over time. In cases of patients with Long Covid with low IFN-γ secretion (Fig. 2B), it may be the case that those patients donated at a nadir in their cycle of IFN-γ secretion, causing a low result. Many of our patients report cyclic symptoms over time.

Although we found that a drop in IFN-γ secretion to baseline levels correlated with symptom resolution for acute COVID-19 (Figs. 1 and 2) and Long Covid (Fig. 5), the cyclical changes in IFN-γ secretion in patients with Long Covid from Fig. 2B do not appear to correlate with severity of symptoms reported by the patients. That is, as long as IFN-γ secretion was above baseline levels, the magnitude of secretion did not correlate with symptom severity as reported by the patient. One issue is that patient-reported severity of symptoms can be variable and subjective: A patient will say in the clinic that they feel much better but then report a relapse a week after the appointment. It therefore may be the case that there is a time delay between IFN-γ secretion and symptom worsening. Alternatively, IFN-γ secretion over a baseline threshold is indicative of Long Covid symptoms, but that higher levels over that threshold do not necessarily predict worse symptoms.

Notably, although the IFN-γ signature correlated with resolution of Long Covid symptoms (Fig. 5), IFN-γ secretion does not correlate strongly with acute disease severity at day 28 (fig. S4). We believe that severity of acute COVID-19 is caused by a myriad of factors including viral immunopathology and cytokine storms that resolve over time. Twenty-eight days after infection, our earliest time point would be outside the acute infection window for most patients, and this might explain the lack of correlation as well.

In addition to IFN-γ, we also observed, using LEGENDplex assays, a smaller increase in IL-1β, IL-6, TNF-α, GM-CSF, and G-CSF secretion from patients with Long Covid, raising the possibility that other proinflammatory cytokines may be perturbed as well, as has been observed by others at 8 months after infection (*19*). We were able to confirm IL-1β and IFN- γ production by ELISA assays and ruled out TNF-α production. GM-CSF production appeared to be lower than the detectability threshold of the ELISA, leaving results inconclusive. This suggests that other proinflammatory cytokines also play a role in Long Covid pathology, and future work will hopefully focus on their roles as well. Overall, however, we do not see a global dysregulation of T cell functionality; the cells do not appear to be either hyperfunctional or unable to respond to peptides from other viruses (fig. S3).

It would be interesting to understand better the CD8^+^ T cells that are making IFN-γ. However, as evidenced by the FluoroSpot assays, these cells represent a low percentage of the overall population and would be difficult to measure using bulk transcriptomics. We would welcome insights using single-cell RNA sequencing analysis of CD8^+^ T cells in patients with Long Covid, which would help to fully identify and characterize the activation profile of these cells.

At this stage, it is not clear whether IFN-γ is a mediator or a biomarker of Long Covid symptoms. Use of IFN-γ treatment for viral infections such as hepatitis C is associated with symptoms such as fever, diarrhea, headache, chills, nausea, myalgia, and/or fatigue (*20*) as well as psychological symptoms such as depression and anxiety (*21*). As some of the symptoms of IFN-γ therapy are similar to those in patients with Long Covid, it is plausible that aberrant IFN-γ production may be a causative factor. If not, then our findings of IFN-γ secretion make it an important tractable biomarker, at least for fatigue associated with Long Covid, which, if explored in the context of a larger patient cohort, has the potential to reveal pathophysiological basis of Long Covid and lead to the discovery of effective drug therapies.

Dysregulated interferon signaling is a hallmark of SARS-CoV-2 infection and plays a key role in disease severity and progression (*22*, *23*). Our data suggest that Long Covid is associated with IFN-γ secretion that is caused by antigen presentation from CD14^+^ cells (Fig. 3E). The cause of this antigen presentation is not fully clear, but our work links with other models describing the causes of Long Covid (*9*). The first possibility is that viral antigens may be persisting in patients with Long Covid, and there have been observations by others of persisting viral antigens even up to 15 months after infection (*24*–*26*). These antigens may be persisting in our patient cohort and causing IFN-γ secretion. Secondly, our work does not rule out the possibility that autoantigens are stimulating IFN-γ secretion, the presentation of which could be initiated by SARS-CoV-2 infection. The presence of autoantibodies has correlated with the risk of Long Covid (*27*, *28*). Third, some have noticed the reactivation of latent herpesviruses correlates with Long Covid (*29*–*31*); it is plausible that antigens from these coinfections are causing IFN-γ secretion. Notably, some have suggested microclotting as a principle cause of Long Covid (*32*). Although our data do not rule out microclotting playing a role in Long Covid, it does not support microclotting as the only cause of Long Covid.

We find patients with Long Covid tend to show persistent unstimulated IFN-γ secretion, in keeping with their unremitting symptoms. In conjunction with patients’ reports, our findings suggest that SARS-CoV-2 is likely the trigger leading to symptoms. Long Covid has some features similar to generic postviral syndrome; hence, the accumulation of proinflammatory cytokines is suggested as a cause of Long Covid (*33*). IFN-γ secretion was a marker of T cell exhaustion in chronic hepatitis C infections (*34*), although we do not see clear indications of T cell exhaustion in our cohort (fig. S3). Increased CD14^+^ cells and decreased regulatory T cells, as we have observed by whole-blood cell count analysis (fig. S5), may be exacerbating this inflammatory state.

Chronic postviral symptoms occur for other infections including 10% of those infected with SARS-CoV-1 and Middle Eastern respiratory syndrome (*35*–*41*), Epstein-Barr virus, dengue, and influenza (*42*–*46*), regardless of disease severity (*42*). For future work, it is worthwhile investigating whether patients with chronic postviral symptoms also exhibit high IFN-γ secretion, and if this is the case, then it could be a biomarker with wider utility. As both the buildup of inflammatory cytokines in the central nervous system have been proposed as a cause for Long Covid and chronic fatigue syndrome (CFS) (*33*, *47*) and since CFS can be triggered by viral infections (*43*), it will be interesting to see whether IFN-γ secretion is higher in patients with CFS as well.

Last, although we observed that many patients reported recovery from Long Covid symptoms over the study period, the mechanism behind this remains unclear. Why vaccination triggered an alleviation of some symptoms, which has been seen by several groups, remains unanswered. The correlation between symptom resolution and IFN-γ secretion, however, strengthens this phenotype as an interesting biomarker for follow-up work. If SARS-CoV-2 antigens continue to persist in people with Long Covid, triggering an IFN-γ response, then vaccination may be helping to clear this antigen. Alternatively, activation of the immune system by vaccination may allow for expression of programmed cell death protein 1 (PD-1) and other markers to switch the immune system off. We hope that our discoveries will provide a basis for Long Covid treatments and diagnostics in the future.

## MATERIALS AND METHODS

### Ethics and recruitment criteria

All patients gave informed written consent in accordance with the Declaration of Helsinki. Unexposed donor samples (*n* = 54) were recruited by the National Institute for Health Research (NIHR) BioResource Cambridge through the ARIA (Anti-viral Responses in Ageing, CBR53) study (*14*) with ethical approval from the Cambridge Human Biology Research Ethics Committee (HBREC.2014.07). This cohort was recruited before October 2019, and so no participants were exposed to SARS-CoV-2 infections. Participants were excluded from the study if they were being treated with oral or intravenous immunomodulatory drugs (including steroids, tacrolimus, cyclosporins, azathioprines, mycophenolate, methotrexate, ritusimab, and cyclophosphamide) within the past 3 months, undergoing injected anti-TNF treatments for rheumatoid arthritis and anyone receiving current or recent (past 24 months) cancer chemotherapy.

For positive controls, patients were recruited after a positive RT-qPCR result, where ethical approval was obtained from the East of England–Cambridge Central Research Ethics Committee (REC) (“NIHR BioResource” REC ref. 17/EE/0025) as described by Bergamaschi *et al.* (*15*). The COVID-confirmed hospitalized patients [day 28 (*n* = 51), day 90 (*n* = 20), and day 180 (*n* = 40)], were enrolled following admission to Addenbrooke’s Hospital, Royal Papworth, and Cambridge and Peterborough Foundation Trust with a confirmed diagnosis of COVID-19 via a positive RT-qPCR test for SARS-CoV-2 as stated in (*15*). Recruitment of inpatients at Addenbrooke’s Hospital and health care workers was undertaken by the NIHR Cambridge Clinical Research Facility outreach team and the NIHR BioResource research nurse team as stated in (*15*). Informed consent was obtained from all participants. Each participant provided 32 ml of peripheral venous blood collected into a 9-ml sodium citrate tube. Clinical data were collected at clinic visit, and routine laboratory tests and inflammatory cytokine panel were assayed appropriately where clinically relevant.

The Long Covid study patients (*n* = 55) were recruited and consented under the Cambridge COVID-19 NIHR BioResource joint Consent Form (REC National Research Ethics Service number T1gC1) study NBR87. Given the evolving nature of the disease trajectory of Long Covid and its relapsing and remitting plethora of symptoms, patients were recruited on the basis of symptoms that had persisted for at least 5 months after acute COVID-19 that could not be explained by an alternative diagnosis. As some patients were infected before routine testing began, a positive RT-qPCR result, antibody seropositity to N, or a positive IL-2 response to M and N peptides (*13*) was required as proof of SARS-CoV-2 infection. To exclude effects from acute reinfections, any patients who were reinfected during the study period (based on a positive RT-qPCR test) were excluded.

In cases where there was a single, predominant symptom at point of study recruitment, these are listed in table S2. In keeping with the multimodal nature of the disease and symptom pattern, inevitably some cases did not have predominant symptom/s as they reported nonspecific generalized symptoms. Patients were excluded from the study where there was a clear alternative diagnosis either from the history and/or results of clinical investigations were definite for a clear-cut diagnosis, e.g., other infectious illnesses such as cytomegalovirus, rhinovirus, bacterial pneumonia, and urinary tract infections, or if there was no evidence for acute COVID-19 (no positive test, positive serology, or positive T cell response to M and N peptides and no history in keeping with acute COVID-19 symptoms). Long Covid study participants were recruited between 26th of August 2020 and 31st of July 2021 from patients attending the Infectious Diseases led Long Covid clinic at Addenbrooke’s Hospital.

### Serology testing

SARS-CoV-2 serology by multiplex particle–based flow cytometry (Luminex): Recombinant SARS-CoV-2 N, S, and receptor binding domain (RBD) were covalently coupled to distinct carboxylated bead sets (Luminex, Netherlands) to form a three-plex assay. The S protein construct used is S-R/PP (*48*). The RBD protein construct used is described by Stadlbauer *et al.*, (*49*). Beads were first activated with 1-ethyl-3-[3-dimethylaminopropyl]carbodiimide hydrochloride (Thermo Fisher Scientific) in the presence of *N*-hydroxysuccinimide (Thermo Fisher Scientific), according to the manufacturer’s instructions, to form amine-reactive intermediates. The activated bead sets were incubated with the corresponding proteins at a concentration of 50 μg/ml in the reaction mixture for 3 hours at room temperature on a rotator. Beads were washed and stored in a blocking buffer [10 mM phosphate-buffered saline (PBS), 1% bovine serum albumin (BSA), and 0.05% NaN_3_].

The N-, S- and RBD-coupled bead sets were incubated with proband sera at a 1:100 dilution for 1 hours in 96-well filter plates (MultiScreen HTS, Millipore) at room temperature in the dark on a horizontal shaker. Fluids were aspirated with a vacuum manifold, and beads were washed three times with 10 mM PBS/0.05% Tween 20. Beads were incubated for 30 min with a phycoerythrin (PE)-labeled anti-human immunoglobulin G–Fc antibody (Leinco/Biotrend), washed as described above, and resuspended in 100 μl of PBS/Tween 20. They were then analyzed on a Luminex analyzer (Luminex/R&D Systems) using Exponent software V31. Specific binding was reported as mean fluorescence intensities. N protein was provided by L. James. RBD was provided by J. Nathan. Trimeric S was provided by J. Briggs.

### PBMC isolation from patient blood and MACS

PBMCs were isolated from citrated blood samples by layering blood onto Lymphoprep (Axis-Shield, Oslo, Norway) and performing density gradient centrifugation at 1200*g* for 10 min. PBMCs at the interface were collected and washed 2× in PBS.

Positive selection of monocytes was performed using MACS with CD14^+^, CD4^+^, or CD8^+^ microbeads (Miltenyi Biotec) as detailed in the manufacturer’s protocol. Whenever PBMCs, CD4/8/14^+^ cell–depleted PBMCs, or isolated cells were plated for FluoroSpot analysis, cells were resuspended in the same volume (2 × 10^5^ PBMCs per well) and then plated.

### Dual FluoroSpot assays

A total of 2 × 10^5^ PBMCs suspended in TexMACS (Miltenyi Biotec) supplemented with 5% human AB serum (Sigma-Aldrich) were incubated on FluoroSpot plates coated with human IFN-γ and IL-2 antibodies or human IFN-γ, TNF-α, or IL-10 antibodies (FluoroSpot, Mabtech AB, Nacka Strand, Sweden) in duplicate with S open reading frame peptides (final peptide concentration, 2 μg/ml per peptide) or TexMACS-only negative control and positive control mix [containing anti-CD3 (Mabtech AB), SEB, and LPS (all Sigma-Aldrich)] at 37°C in a humidified CO_2_ atmosphere for 48 hours (or 24 hours where indicated). The cells and medium were decanted from the plate, and the assay was developed following the manufacturer’s instructions. Developed plates were read using an AID iSpot reader (Oxford Biosystems, Oxford, UK) and counted using AID EliSpot v7 software (Autoimmun Diagnostika GmbH, Strasberg, Germany) using distinct counting protocols for IFN-γ, TNF-α, IL-10, or IL-2 secretion. Donor results were discounted from further analysis if there was less than 100 spot forming units (SFU) in the positive control relative to the background SFU. Limit of detectability is calculated as one spot per 2 × 10^6^ cells, the upper limit of cells that could be loaded per well. S response data in Fig. 5 were corrected for background cytokine production by subtracting the negative control.

We used a peptide pool for S as recently published (*50*): “A peptide pool was generated using the following: (i) PepTivator SARS-CoV-2 Prot_S containing the sequence domains aa 304-338, 421-475, 492-519, 683-707, 741-770, 785-802, and 885-1273 and S1 N-terminal S1 domain of the surface glycoprotein (“S”) of SARS-Coronavirus 2 (GenBank MN908947.3, Protein QHD43416.1). (ii) The PepTivator SARS-CoV-2 Prot_S1 containing the aa sequence 1–692. The peptides used are 15aa amino acids with 11 amino acid overlaps.”

### Absolute count enumeration of lymphocyte subsets

The absolute number of immune cells present in whole-blood samples was enumerated using Becton Dickinson Trucount tubes (BD Biosciences, Oxford, UK) following the manufacturer’s instructions. Briefly, 50 μl of the EDTA-treated whole-blood samples was stained in the Trucount tube with a premixed antibody cocktail (detailed in table S4) allowing the identification of monocytes, B cells, CD4^+^ and CD8^+^ T cells, T cell memory subsets, and activated T cells and NK cells. Following staining, the red blood cells were lysed, and the cells were fixed using fluorescence-activated cell sorting (FACS) lysing solution (BD Biosciences) and then stored at −80°C until acquisition (*51*). Samples were acquired on a five-laser LSRFortessa (BD Biosciences) with fluorescence minus one controls and single-color compensation controls (AbC Total Antibody Compensation Bead Kit, Thermo Fisher Scientific) used. Samples were analyzed and enumerated using FlowJo software (BD Biosciences) following the gating strategy, and the formula was illustrated in fig. S6. Results were expressed as the number of each immune cell subset per microliter of blood (cells per microliter).

### Measuring IFN-γ secretion by flow cytometry

PBMCs (2.5 × 10^6^) from patients with Long Covid and healthy controls suspended in TexMACS medium (Miltenyi Biotec) were unstimulated or stimulated with a positive-control mix [containing anti-CD3 (Mabtech AB), SEB, PHA, pokeweed mitogen, and LPS (all Sigma-Aldrich)] for 1 hour, then brefeldin A (5 μg/ml) and 2 μM monensin (both from BioLegend) were added, and the cells were incubated overnight at 37°C in a humidified CO_2_ atmosphere. The cells were then washed and stained with a combination of surface antibodies comprising CD3 BV650, CD14 fluorescein isothiocyanate, CD19 BV510 (BioLegend), and LIVE/DEAD fixable Aqua dead cell stain (Thermo Fisher Scientific) at 4°C. The cells were fixed, permeabilized using a FIX & PERM cell permeabilization kit (Thermo Fisher Scientific), and stained intracellularly with CD69 Pacific Blue, CD8 BV570, CD4 BV605, CD134 (OX-40) PE, 4-1BB PE–Cy5, CD40L PerCP-Cy5.5 (BioLegend), and IFN-γ BV786 (BD Biosciences) at 4°C in the dark. Full details of the antibodies used in the assay are provided in table S5. Samples were washed and fixed with FluoroFix buffer (BioLegend) and acquired on a BD LSRFortessa cytometer using FACSDiva software. The data were analyzed using FlowJo software following the gating strategy illustrated in fig. S8. The production of IFN-γ and expression of activation markers on resting and stimulated CD4^+^ and CD8^+^ T cells in healthy controls compared to patients with Long Covid were assessed.

### Measuring cytokine secretion by LEGENDplex

Cytokine secretion between Long Covid and healthy control PBMCs were compared using LEGENDplex COVID-19 Cytokine Storm Panels 1 and 2 (13 plex and 12 plex, catalog nos. 741091 and 741142, respectively) from BioLegend. PBMCs samples from *n* = 10 healthy controls and *n* = 14 patients with Long Covid were used. After 48 hours of incubation, medium was collected and loaded onto Legenplex plates at 1:2 dilution, following the manufacturer’s protocol. Samples were analyzed using a BD Accuri C6 following the manufacturer’s instructions, and cytokine concentrations were calculated against a standard curve provided by the manufacturer using BioLegend’s software (www.biolegend.com/en-us/legendplex). ELISAs were used to confirm cytokine secretion from LEGENDplex assays. For these, we used undiluted medium from PBMCs cultured for 48 hours without stimulation. We measured the following cytokines using ELISAMax kits: TNF-α (catalog no. 430201), GM-CSF (catalog no. 432001), IL-1β (catalog no. 437004), and IFN-γ (catalog no. 430115).

### Data handling

Data were determined to be nonparametric by Shapiro-Wilk analysis. We therefore used nonparametric statistical analysis [Mann-Whitney *U*, Kruskal-Wallis one-way analysis of variance (ANOVA), Wilcoxon signed-rank test] throughout. Prism 9.0.2 was used for statistical analysis and data plotting.

## Supplementary Materials

### This PDF file includes:

NIHR BioResource Collaborators Figs. S1 to S9 Tables S1 to S5 References
