# Supplementary material for: Spontaneous, persistent, T cell–dependent IFN-γ release in patients who progress to Long Covid
Source: Sci Adv. 2024 Feb 21;10(8):eadi9379. doi: 10.1126/sciadv.adi9379 (PMC10881041; doi:10.1126/sciadv.adi9379)
Supplement: Supplementary file 1 — NIHR BioResource Collaborators Figs. S1 to S9 Tables S1 to S5 References [file sciadv.adi9379_sm.pdf]

Science Advances
AAAS

Supplementary Materials for

Spontaneous, persistent, T cell–dependent IFN-γ release in patients who progress to Long Covid

Benjamin A. Krishna *et al.*

Corresponding author: Mark R. Wills, mrw1004@cam.ac.uk; Nyaradzai Sithole, ns519@cam.ac.uk

Sci. Adv. **10**, eadi9379 (2024)
DOI: 10.1126/sciadv.adi9379

This PDF file includes:

NIHR BioResource Collaborators
Figs. S1 to S9
Tables S1 to S5
References

Collaborators:

John Allison[1,2], Heather Biggs[1,3,4]John Bradley[5,6,7,8], Helen Butcher[1,2], Daniela Caputo[1,2], Matt Chandler[1,2], Debbie Clapham-Riley[1,2], Patrick Chinnery[1,3,4], Anne-Maree Dean[1,2], Eleanor Dewhurst[1,2], Christian Fernandez[1,8], Anita Furlong[1,2], Anne George [1,2], Barbara Graves[1,2], Jennifer Gray[1,2], Sabine Hein[1,2], Tasmin Ivers[1,2], Mary Kasanicki[1,9], Nathalie Kingston[1,8], Emma Le Gresley[1,2], Rachel Linger[1,2], Sarah Meloy[1,2], Alexei Moulton[1,2], Francesca Muldoon[1,2], Nigel Ovington[1,8], Roxana Paraschiv[1,8], Sofia Papadia[1,2], Isabel Phelan[1,2], Christopher Penkett[1,8], Venkatesh Ranganath[1,8], Jennifer Sambrook[1,8], Katherine Schon[1,3,10], Hannah Stark[1,2], Kathleen E Stirrups[1,8], Paul Townsend[1,8], Julie von Ziegenweidt[1,2], Neil Walker[1,8], Jennifer Webster[1,2]

1. NIHR BioResource, Cambridge University Hospitals, Cambridge Biomedical Campus, Cambridge, CB2 0QQ, UK.
2. Department of Public Health and Primary Care, School of Clinical Medicine, University of Cambridge, Cambridge Biomedical Campus, Cambridge, UK
3. Department of Clinical Neurosciences, School of Clinical Medicine, University of Cambridge, Cambridge Biomedical Campus, Cambridge, UK
4. Medical Research Council Mitochondrial Biology Unit, Cambridge Biomedical Campus, Cambridge, UK
5. Department of Medicine, University of Cambridge, Cambridge CB2 0QQ, UK
6. National Institute for Health Research (NIHR) Cambridge Biomedical Research Centre, Cambridge CB2 0QQ, UK
7. Department of Renal Medicine, Cambridge University Hospitals NHS Foundation Trust, Cambridge CB2 0QQ, UK
8. Department of Haematology, School of Clinical Medicine, University of Cambridge, Cambridge Biomedical Campus, Cambridge, UK
9. Addenbrooke's Hospital, Cambridge University Hospitals NHS Foundation Trust, Cambridge Biomedical Campus, Cambridge, UK
10. Clinical Genetics, Addenbrooke's Hospital, Cambridge University Hospitals NHS Foundation Trust, Cambridge, UK

Figure S1

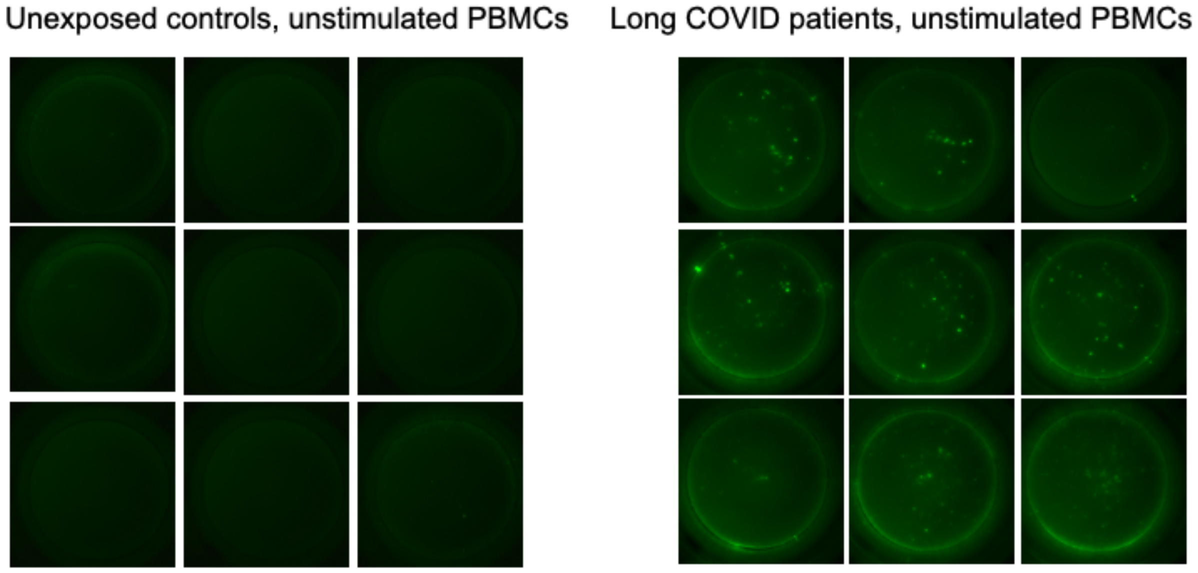

Figure S1: PBMCs from patients with Long COVID produce more interferon gamma. Representative images of wells of unstimulated PBMCs from 9 unexposed donors and 9 patients with Long COVID, taken from the same plate with the same exposures.

Figure S2

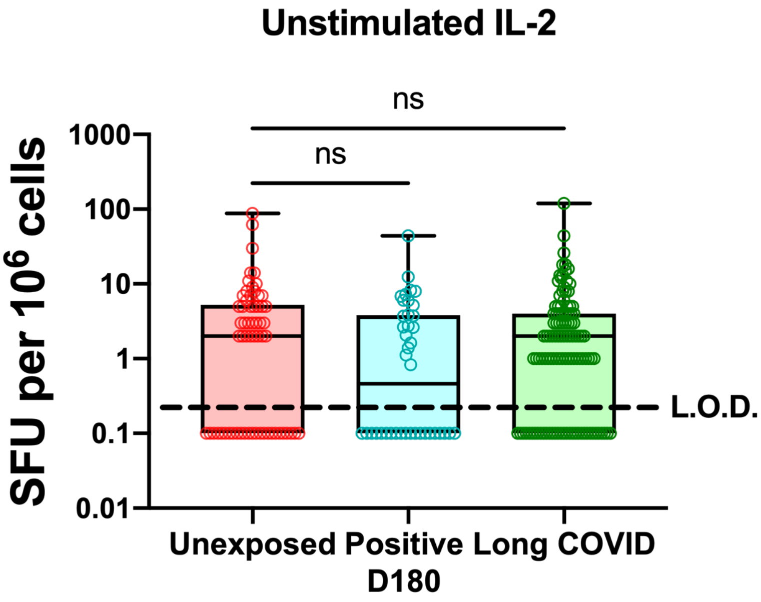

Figure S2: Spontaneous IL-2 release from the PBMCs of Long COVID patients is no different from controls. Data shown is from the same experiment as Figure 1c, having analysed PBMCs from unexposed (red), acutely infected and recovered by D180 (cyan) and Long COVID patients (green). IFN-γ and IL-2 were analysed on the same plates and although a change was seen for IFN-γ, no such change was observed for IL-2. IL-2 release was quantified as spot forming units per million PBMCs. Each donor was run in duplicate and zero results were set as 0.1 to allow their inclusion on a log scale. L.O.D. = limit of detection. Significance calculated by Kruskal-Wallis ANOVA.

Figure S3

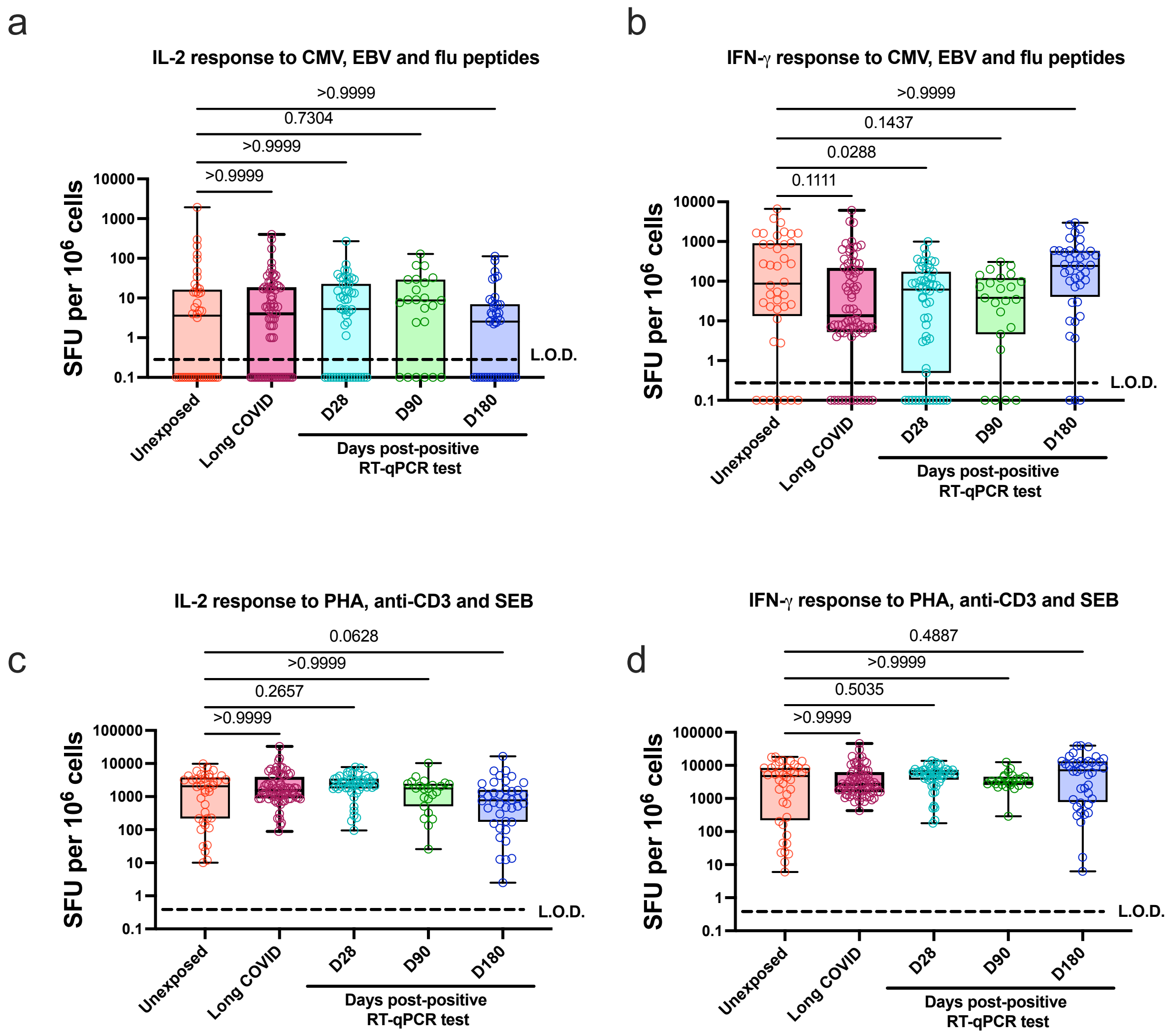

Figure S3: IL-2 and IFN-γ release from peptide- and positive control-stimulated PBMCs show no general T cell disfunction in Long COVID patients. Data shown is from the same cohorts as Figure 1, having analysed PBMCs from unexposed (red), acutely infected and recovered patients at D28 (cyan), D90 (green and D180 (blue) and Long COVID patients (burgundy). These PBMCs were stimulated with either cytomegalovirus (CMV), Epstein-Barr virus (EBV) and influenza virus peptides (CEF) (A&B) or with the positive control cocktail of anti-CD3 antibody, Staphylococcus Enterotoxin B (SEB), and Phytohemagglutinin (PHA) (C&D). IFN-γ and IL-2 responses were measured by FluoroSpot assay as spot forming units per million PBMCs. Each condition was run in duplicate and an unstimulated control was subtracted to remove background cytokine production. Zero results are set as 0.1 to allow their inclusion on a log scale. L.O.D. = limit of detection. Significance calculated by Kruskal-Wallis ANOVA, with Dunn's multiple comparison test between unexposed and each infected group.

Figure S4

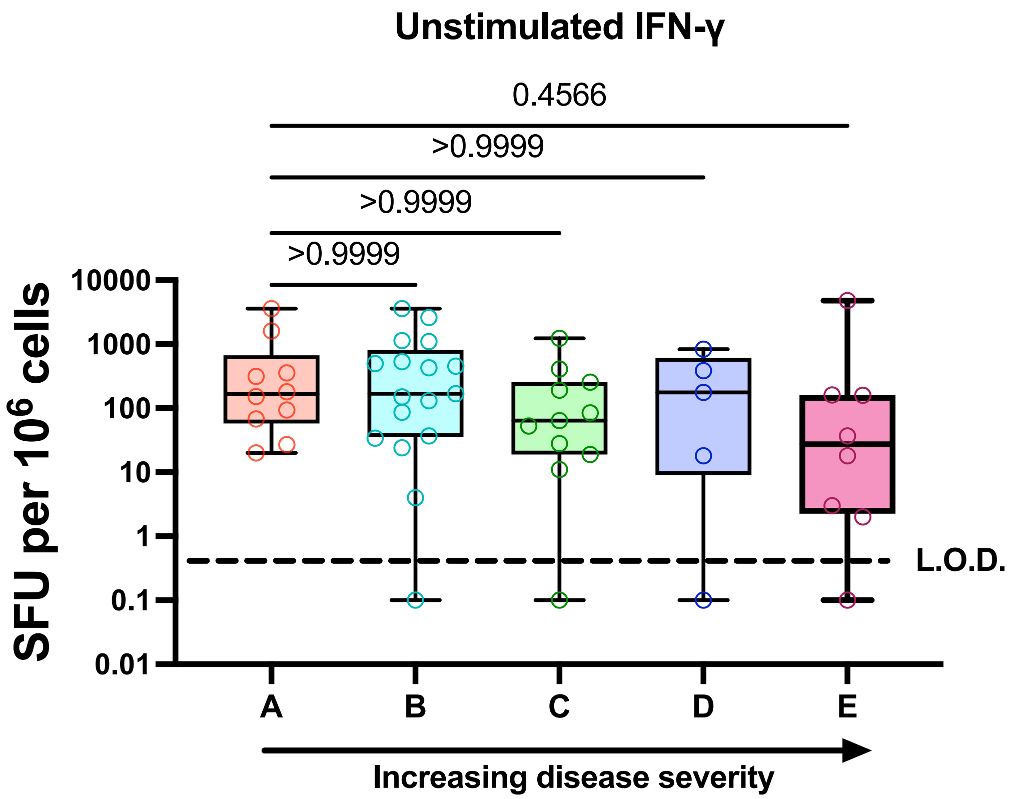

Figure S4: Unstimulated IFN-γ release did not correlate with the severity of acute COVID disease. Data shown is from the acute COVID-19 cohort at D28 in Figure 1B, but the cohort was stratified by disease severity according to [14]. In short, A= asymptomatic, B = symptomatic, C = hospitalised, D = hospitalised with supplemental oxygen and E = hospitalised with assisted ventilation. Zero results are set as 0.1 to allow their inclusion on a log scale. L.O.D. = limit of detection. Significance calculated by Kruskal-Wallis ANOVA, with Dunn's multiple comparison test between group A and each more severe group.

Figure S5

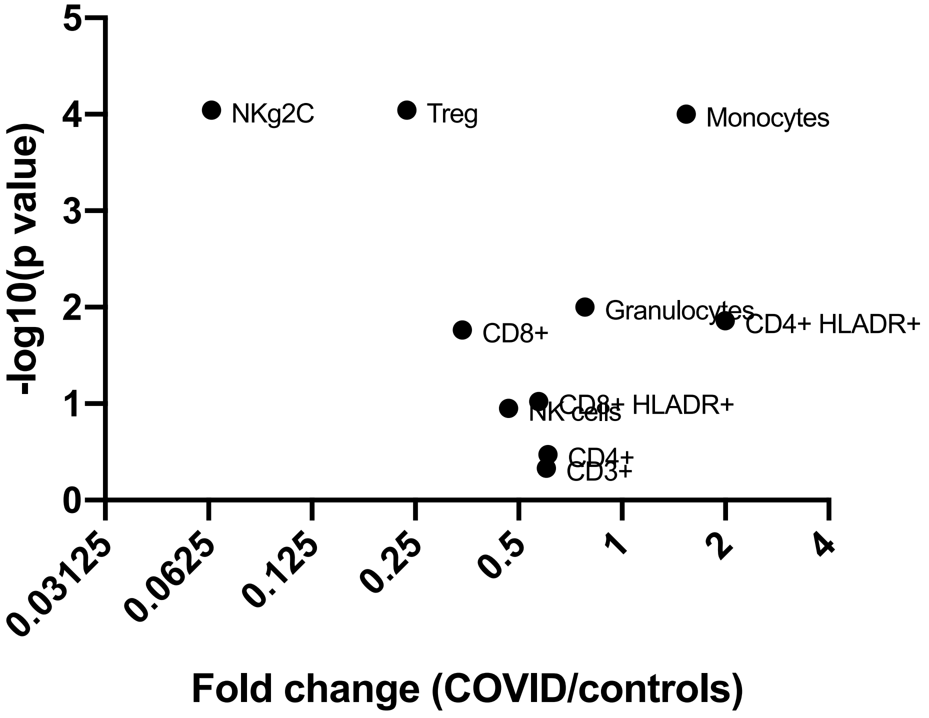

Figure S5: impact of Long COVID on immune cell composition. EDTA treated whole blood was stained with a panel of phenotyping antibodies in order to enumerate T cell, granulocyte, monocyte and NK cell subsets. 18 Long COVID and 26 controls (collected before 2019) were analysed. Data is shown as a volcano plot of fold change in Long COVID patients against healthy controls vs inverse logs of p values, as calculated by multiple Mann-Whitney U tests with Bonferroni-Dunn correction for multiple tests.

Figure S6

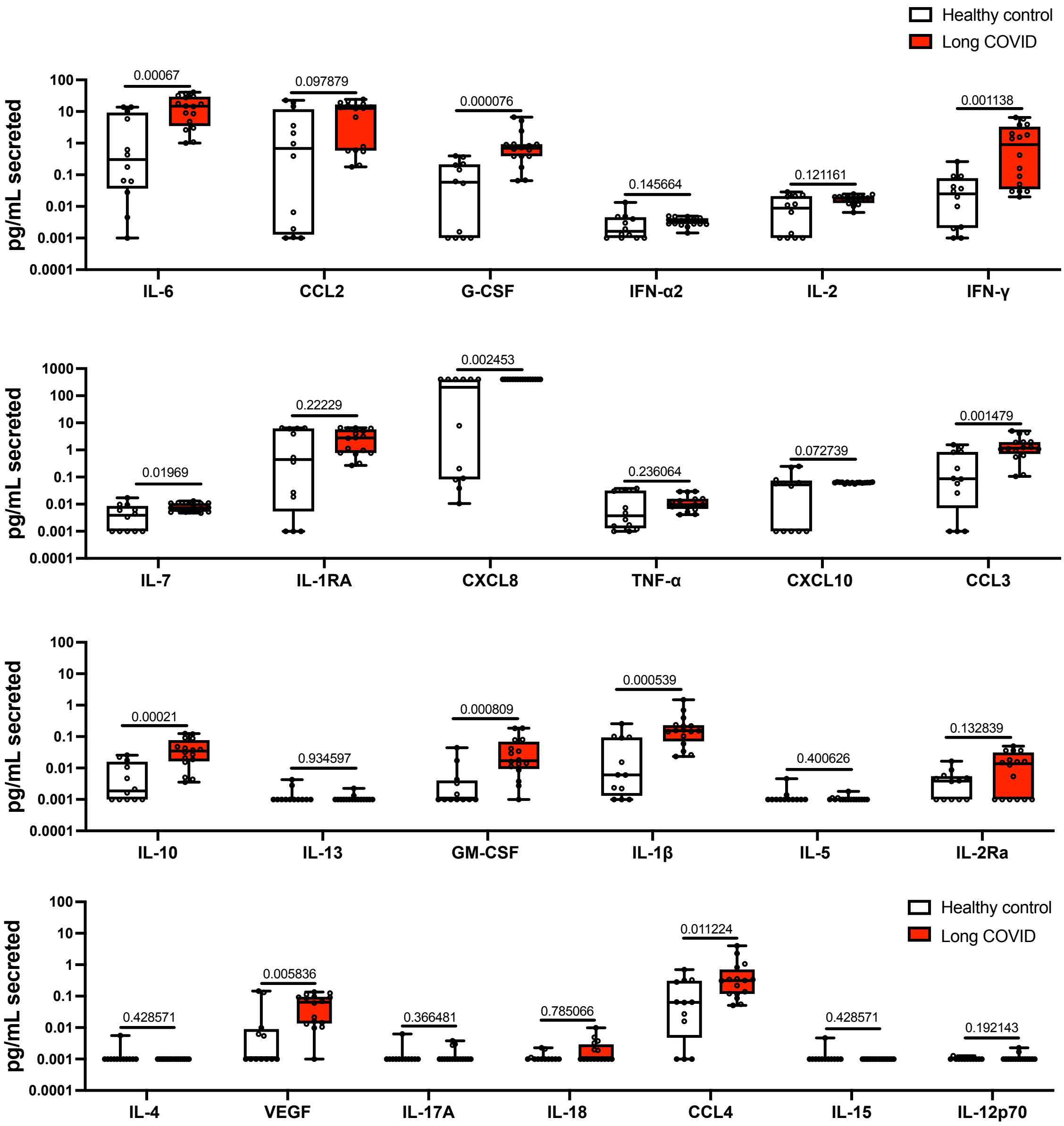
Healthy control
Long COVID
pg/mL secreted
0.00067
0.097879
0.000076
0.145664
0.121161
0.001138
IL-6
CCL2
G-CSF
IFN-α2
IL-2
IFN-γ
0.01969
0.22229
0.002453
0.236064
0.072739
0.001479
IL-7
IL-1RA
CXCL8
TNF-α
CXCL10
CCL3
0.00021
0.934597
0.000809
0.000539
0.400626
0.132839
IL-10
IL-13
GM-CSF
IL-1β
IL-5
IL-2Ra
0.428571
0.005836
0.366481
0.785066
0.011224
0.428571
0.192143
IL-4
VEGF
IL-17A
IL-18
CCL4
IL-15
IL-12p70

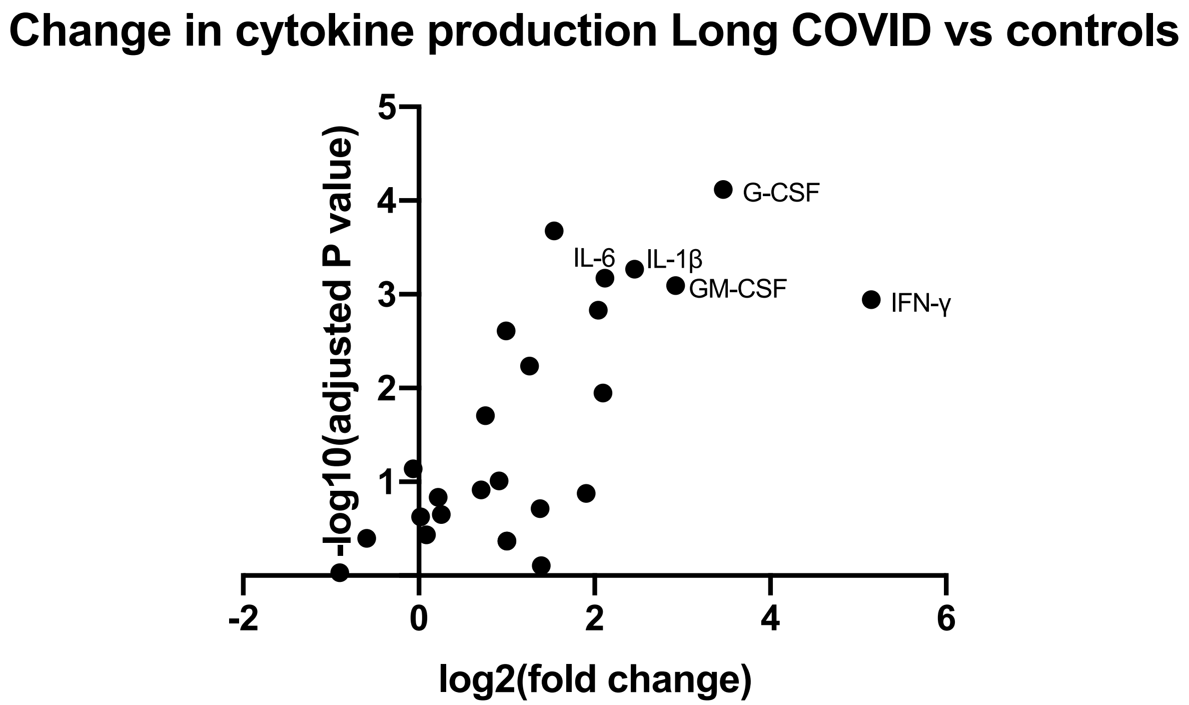

Figure S6: PBMCs from Long COVID spontaneously produce more pro-inflammatory cytokines. PBMCs from patients with Long COVID (n=16) or healthy controls, either unexposed (n=7) or those recovered from acute COVID-19 (n=5), were incubated for 48 hours without exogenous peptide stimulation. Media was collected and cytokine production measured by LEGENDplex assay (Biolegend). The top panel shows the data for each cytokine with individual datapoints, with p values calculated by multiple Mann-Whitney U tests with Bonferroni-Dunn correction for multiple tests. Datapoints below 0.001 pg/mL were set to 0.001 pg/mL to allow visualisation on a logarithmic plot. The bottom panel shows the same data as fold change in cytokine production against p value for each cytokine.

Figure S7

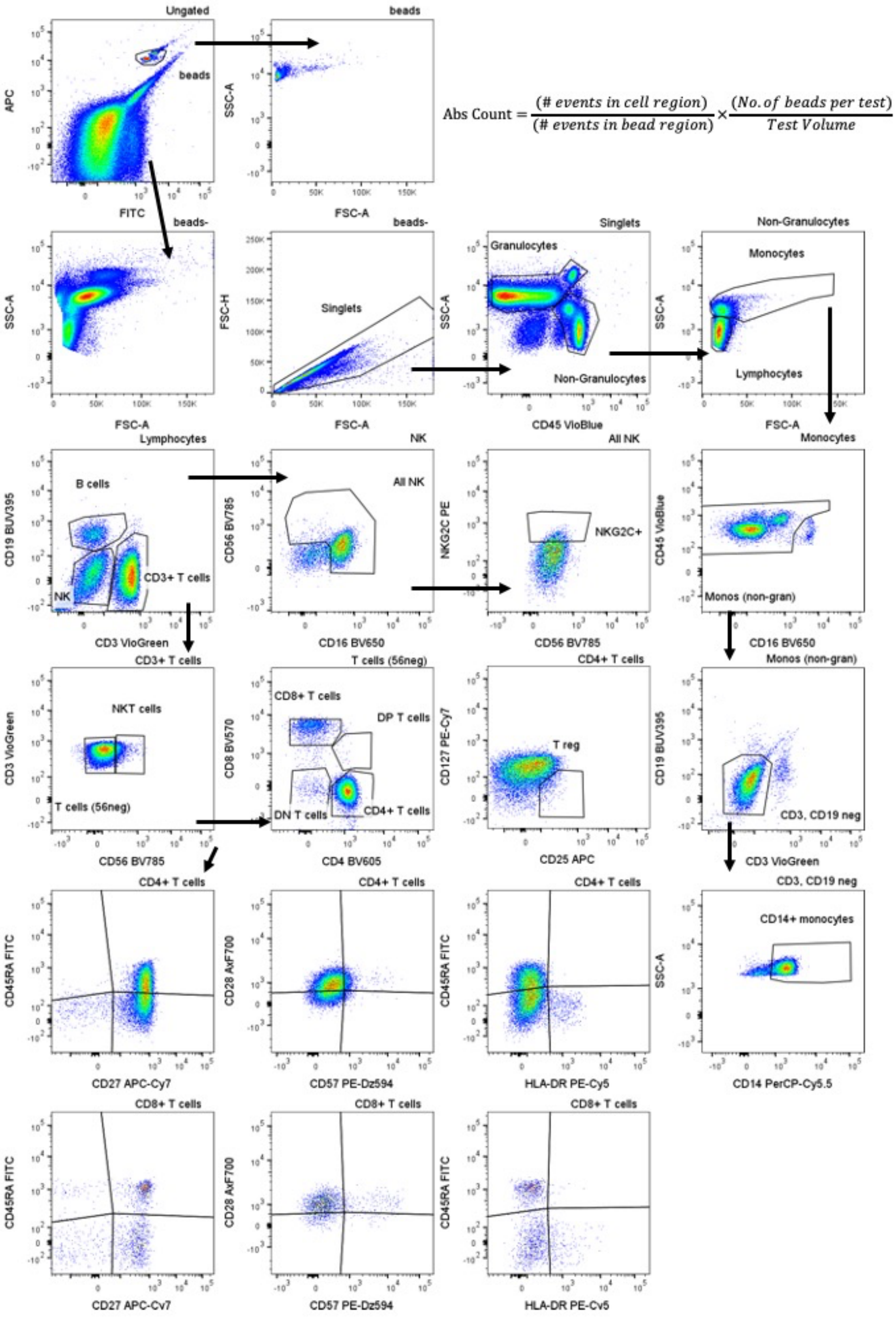
Ungated
beads
APC
FITC
SSC-A
FSC-A
Abs Count = (# events in cell region) / (# events in bead region) × (No. of beads per test) / Test Volume
beads-
FSC-H
Singlets
Granulocytes
Non-Granulocytes
CD45 VioBlue
Monocytes
Lymphocytes
B cells
CD3+ T cells
NK
CD19 BUV395
CD3 VioGreen
All NK
CD56 BV785
CD16 BV650
NKG2C PE
NKG2C+
Monos (non-gran)
CD45 VioBlue
NKT cells
T cells (56neg)
CD8+ T cells
DP T cells
DN T cells
CD4+ T cells
CD8 BV570
CD4 BV605
CD127 PE-Cy7
T reg
CD25 APC
CD3, CD19 neg
CD45RA FITC
CD27 APC-Cy7
CD28 AxF700
CD57 PE-Dz594
HLA-DR PE-Cy5
CD14+ monocytes
CD14 PerCP-Cy5.5

Supplementary Figure 7. Representative dot plots from 1 donor are shown illustrating the gating strategy for generating the absolute count data, as has been used previously [13] [51]. First the trucount bead population was identified (FITC vs APC) and then the trucount bead negative population (i.e. cells) were analysed by gating for single cells (FSC-A vs FSC-H), then CD45^{hi} lymphocytes (Non-Granulocytes) and Granulocytes were gated (CD45 vs SSC-A). Next, a Monocytes gate and Lymphocytes gate were discriminated by size and granularity (FSC-A vs SSC-A) and monocytes were gated by eliminating CD16hi cells (monos (non-gran gate on CD16 vs CD45 plot), the CD3 and CD19 negatives (CD3 vs CD19 plot) and finally the CD14^{+} monocyte population identified (CD14 vs SSC-A). From the lymphocytes gate CD3^{+} T cells, NK cells and B cells were identified (CD3 vs CD19 plot), the NK cells were then gated for CD56^{+}, CD56^{+}CD16^{+} and CD56^{-}CD16^{+} populations as All NK (CD16 vs CD56) and the proportion of NK cells expressing NKG2C was enumerated (CD56 vs NKG2C). The CD3^{+} T cells were gated from the NKT cells (CD56+ by CD56 vs CD3 plot), then the CD4^{+} and CD8^{+} expressing cells and double positive (DP) T cells and double negative (DN) T cells were identified. T regulatory (Treg) CD4^{+} T cells were identified as CD127lo and CD25hi (CD25 vs CD127 plot). Lastly both the CD4^{+} and CD8^{+} T cell populations were further subdivided into 4 memory populations defined by expression of CD27 and CD45RA, 4 differentiation populations defined by expression of CD57 and CD28 and HLA-DR expressing activated cells were identified (HLA-DR vs CD45RA plot). All gate and quadrant positions were identified using the FMO controls, the formula used to calculate the absolute cell counts from the event numbers in each gate or quadrant is illustrated.

Figure S8

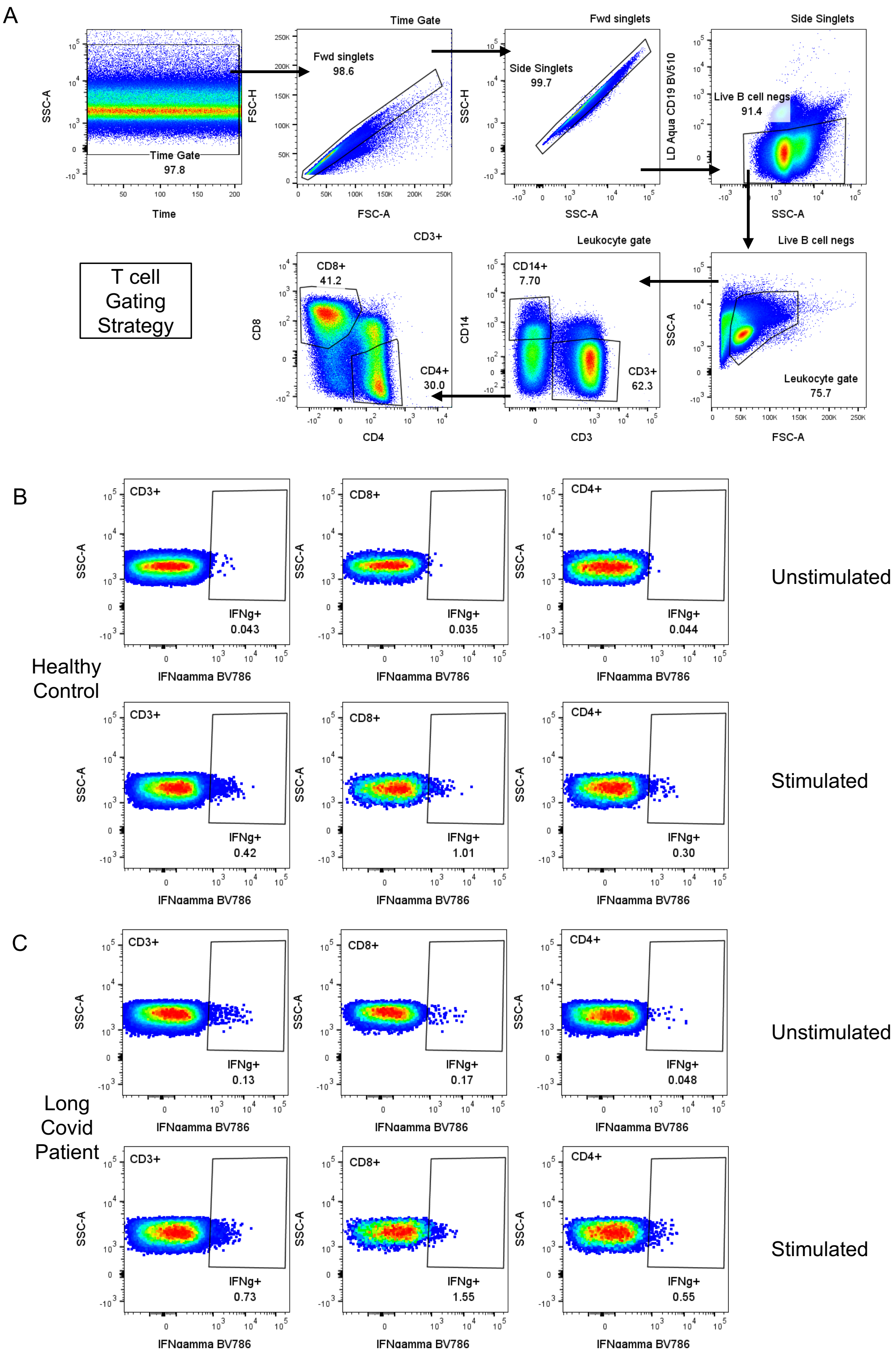

A
SSC-A
Time Gate
97.8
Time
FSC-H
Fwd singlets
98.6
FSC-A
SSC-H
Side Singlets
99.7
SSC-A
LD Aqua CD19 BV510
Live B cell negs
91.4
T cell Gating Strategy
CD3+
CD8
CD8+
41.2
CD4+
30.0
CD4
Leukocyte gate
CD14
CD14+
7.70
CD3+
62.3
CD3
Live B cell negs
Leukocyte gate
75.7
FSC-A
B
Healthy Control
CD3+
CD8+
CD4+
IFNg+
0.043
0.035
0.044
IFNgamma BV786
Unstimulated
IFNg+
0.42
1.01
0.30
Stimulated
C
Long Covid Patient
IFNg+
0.13
0.17
0.048
Unstimulated
IFNg+
0.73
1.55
0.55
Stimulated

Figure S8: The gating strategy used to identify CD4$^+$ and CD8$^+$ T cells is shown (A) with representative plots from 1 donor sample. First a Time vs Side scatter gate was drawn, to identify the main flow of cells, then these cells were gated for forward scatter single cells (Forward scatter area (FSC-A) vs Forward scatter height (FSC-H)), side scatter single cells (Side scatter area (SSC-A) vs Side scatter height (SSC-H)), live B cell negative cells (SSC-W vs Live Dead Aqua dye & CD19 BV510), then lymphocytes were gated (FSC-A vs SSC-A (log scale)). CD3 positive cells were identified (CD3 BV650 vs CD14 FITC) and then CD8$^+$ and CD4$^+$ T cells gated (CD4 BV605 vs CD8 BV570). Representative plots showing IFNγ staining from CD3$^+$ T cells, CD8$^+$ and CD4$^+$ T cell subsets from unstimulated and stimulated (polyclonal positive control) of a Healthy control (B) and long covid patient (C) are shown. This panel has been used before [52, 53]. *This illustrates the increased production of IFNgamma in ex vivo long covid patient T cells compared to the healthy control volunteer.*

Figure S9

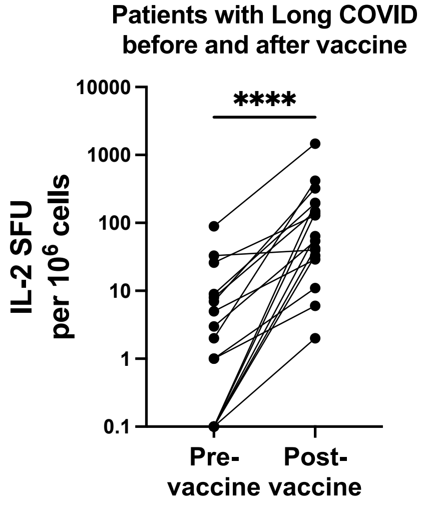

Figure S9: IL-2 is produced in response to spike peptide stimulation after vaccination in patients with Long COVID. PBMCs were isolated from patients diagnosed with Long COVID before and after first vaccination and either stimulated with anti-spike peptide *ex-vivo* or mock treated with a media control. After 48 hours incubation IL-2 release from spike peptide-stimulated T cells was measured by FluoroSpot analysis, with negative control IL-2 subtracted from spike stimulated samples. For both datasets, IL-2 and IFN-γ were quantified as spot forming units per million PBMCs. Each donor was run in duplicate and zero results were set as 0.1 to allow their inclusion on a log scale. L.O.D. = limit of detection. Significance calculated by Wilcoxon Ranked Sign Test, ****$p<0.0001$.

Table S1:

	Unexposed negatives	q-PCR confirmed positives	Long COVID
N	54	81	55
Age, median (IQR)	48 (34-74)	52 (35-64)	46 (35-59.5)
18–30 years	4/54 (7.4%)	10/81 (12%)	8/55 (14.5%)
31–45 years	23/54 (42%)	18/81 (22%)	18/55 (32.7%)
46–60 years	7/54 (13%)	24/81 (29%)	15/55 (27.2%)
>60 years	20/54 (37%)	27/81 (33%)	14/55 (25.4%)
% male	27/54 (50%)	42/81 (52%)	(24/55) 43.6%
%PCR positive	0/54 (0%)	81/81 (100%)	(10/55) 18.2%
% seropositive (both anti-S and anti-N)	n/a	31/81 (38%)	22/55 (40%)
% seronegative	n/a	7/81 (8.6%)	28/55 (51%)
%hospitalised	n/a	24/81 (30%)	7/55 (13%)
%mild illness	n/a	57/81 (70%)	48/55 (87%)
Comorbidities			
Hypertension	n/a	14/81 (17%)	7/55 (12.7%)
Diabetes Mellitus	n/a	13/81 (16%)	1/55 (1.8%)
COPD, Asthma	n/a	7/81 (8.6%)	14/55 (25%)
Anxiety/Depression	n/a	n/a	2/55 (3.6%)
Cancer/Immunosuppression	n/a	3/81 (3.7%)	3/55 (5.4%)
Obesity	n/a	12/81 (15%)	1/55 (1.8%)
Chronic Heart Disease	n/a	8/81 (10%)	1/55 (1.8%)
Time since symptoms (median +/- IQR)	n/a	n/a*	7 (5-12)

n/a – not applicable, used where data was not recorded
n/a* - samples were collected at 28, 90 and 180 days after positive qPCR result

Table S2: Long COVID symptoms distribution post vaccination

Symptom	Number patients reported
Fatigue	48
Shortness of breath	8
Altered taste/smell	4
Palpitations/tachycardia	4
Fever	3
Chest pains	2
Headache	2
Myocarditis	1
Parasthesia	1
Voice change	1
Acid reflux	1

Table S3: Long COVID symptoms distribution post vaccination

	**Long COVID patients**	**Non-hospitalised**	**Hospitalised**	**SARS-CoV-2 antibody positive (negative)**	**IFN-γ status pre-vaccination (post vaccination) (above upper quartile unexposed control)**
**Age categories**					
31-45	5	5	0	1 (4)	5 (4)
46-60	6	6	0	1 (5)	5 (0)
>60	6	2	4	2 (4)	6 (4)
**Gender**					
Female	10	9	3	1 (9)	10 (4)
Male	7	4	3	2 (1)	6 (4)

Table S4: Absolute count antibody panel

Antigen	Fluorochrome	Clone	Isotype	Cat. No.	Supplier
CD19	BUV395	SJ25C1	IgG1 κ	563549	BD BioSciences
CD45	VioBlue	REA747	rh IgG1	130-110-637	Miltenyi Biotec
CD3	VioGreen	REA613	rh IgG1	130-113-142	Miltenyi Biotec
CD8	BV570	RPA-T8	IgG1 κ	301038	BioLegend
CD4	BV605	OKT4	IgG2b κ	317438	BioLegend
CD16	BV650	3G8	IgG1 κ	302042	BioLegend
CD56	BV785	5.1H11	IgG1 κ	362550	BioLegend
CD45RA	FITC	HI100	IgG2b κ	304106	BioLegend
CD14	PerCP-Cy5.5	M5E2	IgG2a κ	301824	BioLegend
NKG2C	PE	REA205	rh IgG1	130-119-776	Miltenyi Biotec
CD57	PE-Dz594	HNK-1	IgM κ	359620	BioLegend
HLA-DR	PE-Cy5	L243	IgG2a κ	307608	BioLegend
CD127	PE-Cy7	A019D5	IgG1 κ	351320	BioLegend
CD25	APC	BC96	IgG1 κ	302610	BioLegend
CD28	AxF700	CD28.2	IgG1 κ	302920	BioLegend
CD27	APC-Cy7	O323	IgG1 κ	302816	BioLegend
CD69	Pacific Blue	FN50	IgG1 κ	310920	BioLegend

Abbrv: BV = Brilliant Violet; BUV = Brilliant UV; Dz = Dazzle; AxF = Alexa Fluor

Antibodies used for flow cytometry were validated for flow cytometry by Biolegend and Miltenyi biotech. Additionally, these antibodies have been used previously by Dr Sarah Jackson for other studies [13, 53, 54].

Table S5: IFN-γ secretion Flow cytometry panel

Antigen	Fluorochrome	Clone	Isotype	Cat. No.	Supplier
CD14	FITC	M5E2	IgG2a κ	301804	BioLegend
CD19	BV510	HIB19	IgG1 κ	302242	BioLegend
CD3	BV650	OKT3	IgG2a κ	317324	BioLegend
CD69	Pacific Blue	FN50	IgG1 κ	310920	BioLegend
CD8	BV570	RPA-T8	IgG1 κ	301038	BioLegend
CD4	BV605	OKT4	IgG2b κ	317438	BioLegend
IFN-γ	BV786	4S.B3	IgG1 κ	563731	BD Biosciences
CD134 (OX-40)	PE	Ber-Act35	IgG1 κ	350004	BioLegend
CD137 (4-1BB)	PE-Cy5	4B4-1	IgG1 κ	309808	BioLegend
CD40L	PerCP-Cy5.5	24-31	IgG1 κ	310834	BioLegend

Antibodies used for flow cytometry were validated for flow cytometry by Biolegend and Miltenyi biotech. Additionally, these antibodies have been used previously by Dr Sarah Jackson for other studies [13, 53, 54].

REFERENCES AND NOTES

1. M. Scherlinger, R. Felten, F. Gallais, C. Nazon, E. Chatelus, L. Pijnenburg, A. Mengin, A. Gras, P. Vidailhet, R. Arnould-Michel, S. Bibi-Triki, R. Carapito, S. Trouillet-Assant, M. Perret, A. Belot, S. Bahram, L. Arnaud, J. E. Gottenberg, S. Fafi-Kremer, J. Sibilia, Refining "long-COVID" by a prospective multimodal evaluation of patients with long-term symptoms attributed to SARS-CoV-2 infection. *Infect. Dis. Ther.* **10**, 1747–1763 (2021).

2. C. Huang, L. Huang, Y. Wang, X. Li, L. Ren, X. Gu, L. Kang, L. Guo, M. Liu, X. Zhou, J. Luo, Z. Huang, S. Tu, Y. Zhao, L. Chen, D. Xu, Y. Li, C. Li, L. Peng, Y. Li, W. Xie, D. Cui, L. Shang, G. Fan, J. Xu, G. Wang, Y. Wang, J. Zhong, C. Wang, J. Wang, D. Zhang, B. Cao, 6-month consequences of COVID-19 in patients discharged from hospital: A cohort study. *Lancet.* **397**, 220–232 (2021).

3. D. Ayoubkhani, K. Khunti, V. Nafilyan, T. Maddox, B. Humberstone, I. Diamond, A. Banerjee, Post-covid syndrome in individuals admitted to hospital with covid-19: Retrospective cohort study. *BMJ* **372**, n693 (2021).

4. A. Gupta, M. V. Madhavan, K. Sehgal, N. Nair, S. Mahajan, T. S. Sehrawat, B. Bikdeli, N. Ahluwalia, J. C. Ausiello, E. Y. Wan, D. E. Freedberg, A. J. Kirtane, S. A. Parikh, M. S. Maurer, A. S. Nordvig, D. Accili, J. M. Bathon, S. Mohan, K. A. Bauer, M. B. Leon, H. M. Krumholz, N. Uriel, M. R. Mehra, M. S. V. Elkind, G. W. Stone, A. Schwartz, D. D. Ho, J. P. Bilezikian, D. W. Landry, Extrapulmonary manifestations of COVID-19. *Nat. Med.* **26**, 1017–1032 (2020).

5. E. J. Thompson, D. M. Williams, A. J. Walker, R. E. Mitchell, C. L. Niedzwiedz, T. C. Yang, C. F. Huggins, A. S. F. Kwong, R. J. Silverwood, G. di Gessa, R. C. E. Bowyer, K. Northstone, B. Hou, M. J. Green, B. Dodgeon, K. J. Doores, E. L. Duncan, F. M. K. Williams, OpenSAFELY Collaborative, A. J. Walker, B. MacKenna, P. Inglesby, C. T. Rentsch, H. J. Curtis, C. E. Morton, J. Morley, A. Mehrkar, S. Bacon, G. Hickman, C. Bates, R. Croker, D. Evans, T. Ward, J. Cockburn, S. Davy, K. Bhaskaran, A. Schultze, E. J. Williamson, W. J. Hulme, H. I. McDonald, L. Tomlinson, R. Mathur, R. M. Eggo, K. Wing, A. Y. S. Wong, H. Forbes, J. Tazare, J. Parry, F. Hester, S. Harper, I. J. Douglas, S. J. W. Evans, L. Smeeth, B. Goldacre, A. Steptoe, D. J. Porteous, R. R. C. McEachan, L. Tomlinson, B. Goldacre, P. Patalay,

G. B. Ploubidis, S. V. Katikireddi, K. Tilling, C. T. Rentsch, N. J. Timpson, N. Chaturvedi, C. J. Steves, Long COVID burden and risk factors in 10 UK longitudinal studies and electronic health records. *Nat. Commun.* **13**, 3528 (2022).

6. J. K. Logue, N. M. Franko, D. J. McCulloch, D. McDonald, A. Magedson, C. R. Wolf, H. Y. Chu, Sequelae in adults at 6 months after COVID-19 infection. *JAMA Netw. Open* **4**, e210830-e210830 (2021).

7. M. S. Petersen, M. F. Kristiansen, K. D. Hanusson, M. E. Danielsen, B. Á. Steig, S. Gaini, M. Strøm, P. Weihe, Long COVID in the Faroe Islands-A longitudinal study among non-hospitalized patients. *Clin. Infect. Dis.* **73**, e4058–e4063 (2021).

8. S. M. LaVergne, S. Stromberg, B. A. Baxter, T. L. Webb, T. S. Dutt, K. Berry, M. Tipton, J. Haberman, B. R. Massey, K. McFann, O. Alnachoukati, L. Zier, T. Heacock, G. D. Ebel, M. Henao-Tamayo, J. Dunn, E. P. Ryan, A longitudinal SARS-CoV-2 biorepository for COVID-19 survivors with and without post-acute sequelae. *BMC Infect. Dis.* **21**, 677 (2021).

9. B. Krishna, M. Wills, N. Sithole, Long COVID: What is known and what gaps need to be addressed. *Br. Med. Bull.* **147**, 6–19 (2023).

10. L. Townsend, J. Dowds, K. O'Brien, G. Sheill, A. H. Dyer, B. O'Kelly, J. P. Hynes, A. Mooney, J. Dunne, C. Ni Cheallaigh, C. O'Farrelly, N. M. Bourke, N. Conlon, I. Martin-Loeches, C. Bergin, P. Nadarajan, C. Bannan, Persistent poor health after COVID-19 is not associated with respiratory complications or initial disease severity. *Ann. Am. Thorac. Soc.* **18**, 997–1003 (2021).

11. W. D. Strain, O. Sherwood, A. Banerjee, V. Van der Togt, L. Hishmeh, J. Rossman, The impact of COVID vaccination on symptoms of Long COVID. An international survey of people with lived experience of Long COVID. *Vaccines*. **10**, 652 (2022).

12. B. A. Krishna, M. Metaxaki, M. R. Wills, N. Sithole, Reduced incidence of long coronavirus disease referrals to the Cambridge University teaching hospital long coronavirus disease clinic. *Clin. Infect. Dis.* **76**, 738–740 (2023).

13. B. A. Krishna, E. Y. Lim, L. Mactavous, NIHR BioResource Team, P. A. Lyons, R. Doffinger, J. R. Bradley, K. G. C. Smith, J. Sinclair, N. J. Matheson, P. J. Lehner, M. R. Wills, N. Sithole, Evidence of previous SARS-CoV-2 infection in seronegative patients with Long COVID. *EBioMedicine* **81**, 104129 (2022).

14. S. E. Jackson, *G. X. Sedikides, G. Okecha, E. L. Poole, J. H. Sinclair, M. R. Wills*, Latent cytomegalovirus (CMV) infection does not detrimentally alter T cell responses in the healthy old, but increased latent CMV carriage is related to expanded CMV-specific T cells. *Front. Immunol.* **8**, 733 (2017).

15. L. Bergamaschi, F. Mescia, L. Turner, A. L. Hanson, P. Kotagiri, B. J. Dunmore, H. Ruffieux, A. de Sa, O. Huhn, M. D. Morgan, P. P. Gerber, M. R. Wills, S. Baker, F. J. Calero-Nieto, R. Doffinger, G. Dougan, A. Elmer, I. G. Goodfellow, R. K. Gupta, M. Hosmillo, K. Hunter, N. Kingston, P. J. Lehner, N. J. Matheson, J. K. Nicholson, A. M. Petrunkina, S. Richardson, C. Saunders, J. E. D. Thaventhiran, E. J. M. Toonen, M. P. Weekes, Cambridge Institute of Therapeutic Immunology and Infectious Disease-National Institute of Health Research (CITIID-NIHR) COVID BioResource Collaboration, B. Göttgens, M. Toshner, C. Hess, J. R. Bradley, P. A. Lyons, K. G. C. Smith, Longitudinal analysis reveals that delayed bystander CD8+ T cell activation and early immune pathology distinguish severe COVID-19 from mild disease. *Immunity* **54**, 1257–1275.e8 (2021).

16. J. K. Files, *S. Sarkar, T. R. Fram, S. Boppana, S. Sterrett, K. Qin, A. Bansal, D. M. Long, S. Sabbaj, J. J. Kobie, P. A. Goepfert, N. Erdmann*, Duration of post–COVID-19 symptoms are associated with sustained SARS-CoV-2 specific immune responses. *JCI insight* **6**, e151544 (2021).

17. C. H. Sudre, B. Murray, T. Varsavsky, M. S. Graham, R. S. Penfold, R. C. Bowyer, J. C. Pujol, K. Klaser, M. Antonelli, L. S. Canas, E. Molteni, M. Modat, M. Jorge Cardoso, A. May, S. Ganesh, R. Davies, L. H. Nguyen, D. A. Drew, C. M. Astley, A. D. Joshi, J. Merino, N. Tsereteli, T. Fall, M. F. Gomez, E. L. Duncan, C. Menni, F. M. K. Williams, P. W. Franks, A. T. Chan, J. Wolf, S. Ourselin, T. Spector, C. J. Steves, Attributes and predictors of Long COVID. *Nat. Med.* **27**, 626–631 (2021).

18. M. Taquet, Q. Dercon, S. Luciano, J. R. Geddes, M. Husain, P. J. Harrison, Incidence, co-occurrence, and evolution of long-COVID features: A 6-month retrospective cohort study of 273,618 survivors of COVID-19. *PLoS Med.* **18**, e1003773 (2021).

19. C. Phetsouphanh, D. R. Darley, D. B. Wilson, A. Howe, C. M. L. Munier, S. K. Patel, J. A. Juno, L. M. Burrell, S. J. Kent, G. J. Dore, A. D. Kelleher, G. V. Matthews, Immunological dysfunction persists for 8 months following initial mild-to-moderate SARS-CoV-2 infection. *Nat. Immunol.* **23**, 210–216 (2022).

20. C. H. T. Miller, S. G. Maher, H. A. Young, Clinical use of interferon-γ. *Ann. N. Y. Acad. Sci.* **1182**, 69–79 (2009).

21. E. F. Pinto, C. Andrade, Interferon-related depression: A primer on mechanisms, treatment, and prevention of a common clinical problem. *Curr. Neuropharmacol.* **14**, 743–748 (2016).

22. D. Acharya, G. Liu, M. U. Gack, Dysregulation of type I interferon responses in COVID-19. *Nat. Rev. Immunol.* **20**, 397–398 (2020).

23. COVID-19 Host Genetics Initiative, Mapping the human genetic architecture of COVID-19. *Nature*. **600**, 472–477 (2021).

24. J. García-Abellán, S. Padilla, M. Fernández-González, J. A. García, V. Agulló, M. Andreo, S. Ruiz, A. Galiana, F. Gutiérrez, M. Masiá, Antibody response to SARS-CoV-2 is associated with long-term clinical outcome in patients with COVID-19: A longitudinal study. *J. Clin. Immunol.*, (2021), **41**, 1490, 1501.

25. B. K. Patterson E. B. Francisco, R. Yogendra, E. Long, A. Pise, H. Rodrigues, E. Hall, M. Herrera, P. Parikh, J. Guevara-Coto, T. J. Triche, P. Scott, S. Hekmati, D. Maglinte, X. Chang, R. A. Mora-Rodríguez, J. Mora, Persistence of SARS CoV-2 S1 protein in CD16+ monocytes in post-acute sequelae of COVID-19 (PASC) up to 15 months post-infection. *Front. Immunol.* **12**, 746021 (2022).

26. A. Carmo, J. Pereira-Vaz, V. Mota, A. Mendes, C. Morais, A. C. da Silva, E. Camilo, C. S. Pinto, E. Cunha, J. Pereira, M. Coucelo, P. Martinho, L. Correia, G. Marques, L. Araújo, F.

Rodrigues, Clearance and persistence of SARS-CoV-2 RNA in patients with COVID-19. *J. Med. Virol.* **92**, 2227–2231 (2020).

27. E. Y. Wang, T. Mao, J. Klein, Y. Dai, J. D. Huck, J. R. Jaycox, F. Liu, T. Zhou, B. Israelow, P. Wong, A. Coppi, C. Lucas, J. Silva, J. E. Oh, E. Song, E. S. Perotti, N. S. Zheng, S. Fischer, M. Campbell, J. B. Fournier, A. L. Wyllie, C. B. F. Vogels, I. M. Ott, C. C. Kalinich, M. E. Petrone, A. E. Watkins, Yale IMPACT Team, A. Obaid, A. J. Moore, A. Casanovas-Massana, A. Lu-Culligan, A. Nelson, A. Nunez, A. Martin, B. Geng, C. D. Odio, C. A. Harden, C. Todeasa, C. Jensen, D. Kim, D. McDonald, D. Shepard, E. Courchaine, E. B. White, E. Silva, E. Kudo, G. DeIuliis, H. Rahming, H. J. Park, I. Matos, J. Nouws, J. Valdez, J. Lim, K. A. Rose, K. Anastasio, K. Brower, L. Glick, L. Sharma, L. Sewanan, L. Knaggs, M. Minasyan, M. Batsu, M. Kuang, M. Nakahata, M. Linehan, M. H. Askenase, M. Simonov, M. Smolgovsky, N. Sonnert, N. Naushad, P. Vijayakumar, R. Martinello, R. Datta, R. Handoko, S. Bermejo, S. Prophet, S. Bickerton, S. Velazquez, T. Rice, W. Khoury-Hanold, X. Peng, Y. Yang, Y. Cao, Y. Strong, C. dela Cruz, S. F. Farhadian, W. L. Schulz, S. Ma, N. D. Grubaugh, A. I. Ko, A. Iwasaki, A. M. Ring, Diverse functional autoantibodies in patients with COVID-19. *Nature* **595**, 283–288 (2021).

28. A. Nalbandian, K. Sehgal, A. Gupta, M. V. Madhavan, C. McGroder, J. S. Stevens, J. R. Cook, A. S. Nordvig, D. Shalev, T. S. Sehrawat, N. Ahluwalia, B. Bikdeli, D. Dietz, C. der-Nigoghossian, N. Liyanage-Don, G. F. Rosner, E. J. Bernstein, S. Mohan, A. A. Beckley, D. S. Seres, T. K. Choueiri, N. Uriel, J. C. Ausiello, D. Accili, D. E. Freedberg, M. Baldwin, A. Schwartz, D. Brodie, C. K. Garcia, M. S. V. Elkind, J. M. Connors, J. P. Bilezikian, D. W. Landry, E. Y. Wan, Post-acute COVID-19 syndrome. *Nat. Med.* **27**, 601–615 (2021).

29. Y. Su, D. Yuan, D. G. Chen, R. H. Ng, K. Wang, J. Choi, S. Li, S. Hong, R. Zhang, J. Xie, S. A. Kornilov, K. Scherler, A. J. Pavlovitch-Bedzyk, S. Dong, C. Lausted, I. Lee, S. Fallen, C. L. Dai, P. Baloni, B. Smith, V. R. Duvvuri, K. G. Anderson, J. Li, F. Yang, C. J. Duncombe, D. McCulloch, C. Rostomily, P. Troisch, J. Zhou, S. Mackay, Q. DeGottardi, D. H. May, R. Taniguchi, R. M. Gittelman, M. Klinger, T. M. Snyder, R. Roper, G. Wojciechowska, K. Murray, R. Edmark, S. Evans, L. Jones, Y. Zhou, L. Rowen, R. Liu, W. Chour, H. A. Algren, W. R. Berrington, J. A. Wallick, R. A. Cochran, M. E. Micikas, ISB-Swedish COVID-19

Biobanking Unit, T. Wrin, C. J. Petropoulos, H. R. Cole, T. D. Fischer, W. Wei, D. S. B. Hoon, N. D. Price, N. Subramanian, J. A. Hill, J. Hadlock, A. T. Magis, A. Ribas, L. L. Lanier, S. D. Boyd, J. A. Bluestone, H. Chu, L. Hood, R. Gottardo, P. D. Greenberg, M. M. Davis, J. D. Goldman, J. R. Heath, Multiple early factors anticipate post-acute COVID-19 sequelae. *Cell*, (2022), **185**, 881, 895.e20.

30. J. Klein, J. Wood, J. R. Jaycox, R. M. Dhodapkar, P. Lu, J. R. Gehlhausen, A. Tabachnikova, K. Greene, L. Tabacof, A. A. Malik, V. S. Monteiro, J. Silva, K. Kamath, M. Zhang, A. Dhal, I. M. Ott, G. Valle, M. Peña-Hernández, T. Mao, B. Bhattacharjee, T. Takahashi, C. Lucas, E. Song, D. M. Carthy, E. Breyman, J. Tosto-Mancuso, Y. Dai, E. Perotti, K. Akduman, T. J. Tzeng, L. Xu, A. C. Geraghty, M. Monje, I. Yildirim, J. Shon, R. Medzhitov, D. Lutchmansingh, J. D. Possick, N. Kaminski, S. B. Omer, H. M. Krumholz, L. Guan, C. S. Dela Cruz, D. van Dijk, A. M. Ring, D. Putrino, A. Iwasaki, Distinguishing features of Long COVID identified through immune profiling. *Nature*, **623**, 139–148 (2023).

31. M. J. Peluso, T. M. Deveau, S. E. Munter, D. Ryder, A. Buck, G. Beck-Engeser, F. Chan, S. Lu, S. A. Goldberg, R. Hoh, V. Tai, L. Torres, N. S. Iyer, M. Deswal, L. H. Ngo, M. Buitrago, A. Rodriguez, J. Y. Chen, B. C. Yee, A. Chenna, J. W. Winslow, C. J. Petropoulos, A. N. Deitchman, J. Hellmuth, M. A. Spinelli, M. S. Durstenfeld, P. Y. Hsue, J. D. Kelly, J. N. Martin, S. G. Deeks, P. W. Hunt, T. J. Henrich, Chronic viral coinfections differentially affect the likelihood of developing Long COVID. *J. Clin. Invest.* **133**, e163669 (2023).

32. A. Kruger, M. Vlok, S. Turner, C. Venter, G. J. Laubscher, D. B. Kell, E. Pretorius, Proteomics of fibrin amyloid microclots in Long COVID/post-acute sequelae of COVID-19 (PASC) shows many entrapped pro-inflammatory molecules that may also contribute to a failed fibrinolytic system. *Cardiovasc. Diabetol.* **21**, 190 (2022).

33. R. Perrin, L. Riste, M. Hann, A. Walther, A. Mukherjee, A. Heald, Into the looking glass: Post-viral syndrome post COVID-19. *Med. Hypotheses* **144**, 110055–110055 (2020).

34. C. Cai, J. Samir, M. R. Pirozyan, T. N. Adikari, M. Gupta, P. Leung, B. Hughes, W. van der Byl, S. Rizzetto, A. Elthala, E. Keoshkerian, J. L. Palgen, T. Peters, T. H. O. Nguyen, R. Louie, K. Kedzierska, S. Gaudieri, R. A. Bull, A. R. Lloyd, F. Luciani, Identification of human

progenitors of exhausted $CD8^+$ T cells associated with elevated IFN-γ response in early phase of viral infection. *Nat. Commun.* **13**, 7543 (2022).

35. H. Ahmed, K. Patel, D. C. Greenwood, S. Halpin, P. Lewthwaite, A. Salawu, L. Eyre, A. Breen, R. O'Connor, A. Jones, M. Sivan, Long-term clinical outcomes in survivors of severe acute respiratory syndrome and Middle East respiratory syndrome coronavirus outbreaks after hospitalisation or ICU admission: A systematic review and meta-analysis. *J. Rehabil. Med.* **52**, jrm00063 (2020).

36. D. Hui, G. M. Joynt, K. T. Wong, C. D. Gomersall, T. S. Li, G. Antonio, F. W. Ko, M. C. Chan, D. P. Chan, M. W. Tong, T. H. Rainer, A. T. Ahuja, C. S. Cockram, J. J. Sung, Impact of severe acute respiratory syndrome (SARS) on pulmonary function, functional capacity and quality of life in a cohort of survivors. *Thorax* **60**, 401–409 (2005).

37. M. H.-B. Lam, Y.-K. Wing, Mandy Wai-Man Yu, C.-M. Leung, R. C. W. Ma, A. P. S. Kong, W Y So, Samson Yat-Yuk Fong, S.-P. Lam, Mental morbidities and chronic fatigue in severe acute respiratory syndrome survivors: Long-term follow-up. *Arch. Intern. Med.* **169**, 2142–2147 (2009).

38. A. M. Lee, J. G. W. S. Wong, G. M. McAlonan, V. Cheung, C. Cheung, P. C. Sham, C. M. Chu, P. C. Wong, K. W. T. Tsang, S. E. Chua, Stress and psychological distress among SARS survivors 1 year after the outbreak. *Can. J. Psychiatry* **52**, 233–240 (2007).

39. H. Moldofsky, J. Patcai, Chronic widespread musculoskeletal pain, fatigue, depression and disordered sleep in chronic post-SARS syndrome; a case-controlled study. *BMC Neurol.* **11**, 37 (2011).

40. K. C. Ong, A. W. Ng, L. S. Lee, G. Kaw, S. K. Kwek, M. K. Leow, A. Earnest, Pulmonary function and exercise capacity in survivors of severe acute respiratory syndrome. *Eur. Respir. J.* **24**, 436–442 (2004).

41. S. H. Lee, H. S. Shin, H. Y. Park, J. L. Kim, J. J. Lee, H. Lee, S. D. Won, W. Han, Depression as a mediator of chronic fatigue and post-traumatic stress symptoms in Middle East respiratory syndrome survivors. *Psychiatry Investig.* **16**, 59–64 (2019).

42. R. C. S. Seet, A. M. L. Quek, E. C. H. Lim, Post-infectious fatigue syndrome in dengue infection. *J. Clin. Virol.* **38**, 1–6 (2007).

43. P. D. White, J. M. Thomas, H. O. Kangro, W. D. A. Bruce-Jones, J. Amess, D. H. Crawford, S. A. Grover, A. W. Clare, Predictions and associations of fatigue syndromes and mood disorders that occur after infectious mononucleosis. *Lancet.* **358**, 1946–1954 (2001).

44. D. S. Buchwald, T. D. Rea, W. J. Katon, J. E. Russo, R. L. Ashley, Acute infectious mononucleosis: Characteristics of patients who report failure to recover. *Am. J. Med.* **109**, 531–537 (2000).

45. I. Petersen, J. Thomas, W. Hamilton, P. White, Risk and predictors of fatigue after infectious mononucleosis in a large primary-care cohort. *Qjm* **99**, 49–55 (2006).

46. B. Z. Katz, Y. Shiraishi, C. J. Mears, H. J. Binns, R. Taylor, Chronic fatigue syndrome after infectious mononucleosis in adolescents. *Pediatrics* **124**, 189–193 (2009).

47. L. Hives, A. Bradley, J. Richards, C. Sutton, J. Selfe, B. Basu, K. Maguire, G. Sumner, T. Gaber, A. Mukherjee, R. N. Perrin, Can physical assessment techniques aid diagnosis in people with chronic fatigue syndrome/myalgic encephalomyelitis? A diagnostic accuracy study *BMJ open* **7**, e017521 (2017).

48. X. Xiong, K. Qu, K. A. Ciazynska, M. Hosmillo, A. P. Carter, S. Ebrahimi, Z. Ke, S. H. W. Scheres, L. Bergamaschi, G. L. Grice, Y. Zhang, The CITIID-NIHR COVID-19 BioResource Collaboration, J. Bradley, P. A. Lyons, K. G. C. Smith, M. Toshner, A. Elmer, C. Ribeiro, J. Kourampa, S. Jose, J. Kennet, J. Rowlands, A. Meadows, C. O'Brien, R. Rastall, C. Crucusio, S. Hewitt, J. Price, J. Calder, L. Canna, A. Bucke, H. Tordesillas, J. Harris, V. Ruffolo, J. Domingo, B. Graves, H. Butcher, D. Caputo, E. le Gresley, B. J. Dunmore, J. Martin, E. Legchenko, C. Treacy, C. Huang, J. Wood, R. Sutcliffe, J. Hodgson, J. Shih, S. Graf, Z. Tong, F. Mescia, T.

Tilly, C. O'Donnell, K. Hunter, L. Pointon, N. Pond, M. Wylot, E. Jones, S. Fawke, B. Bullman, L. Bergamaschi, L. Turner, I. Jarvis, O. Omarjee, A. de Sa, J. Marsden, A. Betancourt, M. Perera, M. Epping, N. Richoz, G. Bower, R. Sharma, F. Nice, O. Huhn, H. Stark, N. Walker, K. Stirrups, N. Ovington, E. Dewhust, E. Li, S. Papadia, J. A. Nathan, S. Baker, L. C. James, H. E. Baxendale, I. Goodfellow, R. Doffinger, J. A. G. Briggs, A thermostable, closed SARS-CoV-2 spike protein trimer. *Nat. Struct. Mol. Biol.* **27**, 934–941 (2020).

49. D. Stadlbauer, F. Amanat, V. Chromikova, K. Jiang, S. Strohmeier, G. A. Arunkumar, J. Tan, D. Bhavsar, C. Capuano, E. Kirkpatrick, P. Meade, R. N. Brito, C. Teo, M. McMahon, V. Simon, F. Krammer, SARS-CoV-2 seroconversion in humans: A detailed protocol for a serological assay, antigen production, and test setup. *Curr. Protoc. Microbiol.* **57**, e100 (2020).

50. D. A. Collier, I. A. T. M. Ferreira, P. Kotagiri, R. P. Datir, E. Y. Lim, E. Touizer, B. Meng, A. Abdullahi, CITIID-NIHR Bio Resource COVID- Collaboration, A. Elmer, N. Kingston, B. Graves, E. L. Gresley, D. Caputo, L. Bergamaschi, K. G. C. Smith, J. R. Bradley, L. Ceron-Gutierrez, P. Cortes-Acevedo, G. Barcenas-Morales, M. A. Linterman, L. E. Mc Coy, C. Davis, E. Thomson, P. A. Lyons, E. M. Kinney, R. Doffinger, M. Wills, R. K. Gupta, Age-related immune response heterogeneity to SARS-CoV-2 vaccine BNT162b2. *Nature*, **596**, 417–422 (2021).

51. T. Hensley-McBain, A. Heit, S. C. De Rosa, M. J. McElrath, E. Andersen-Nissen, Optimization of a whole blood phenotyping assay for enumeration of peripheral blood leukocyte populations in multicenter clinical trials. *J. Immunol. Methods* **411**, 23–36 (2014).

52. E. L. Davies, M. Noor, E. Y. Lim, C. J. Houldcroft, G. Okecha, C. Atkinson, M. B. Reeves, S. E. Jackson, M. R. Wills, HCMV carriage in the elderly diminishes anti-viral functionality of the adaptive immune response resulting in virus replication at peripheral sites. *Front. Immunol.* **13**, 1083230 (2022).

53. S. E. Jackson, G. X. Sedikides, V. Romashova, G. Okecha, E. B. M. Remmerswaal, F. J. Bemelman, J. H. Sinclair, M. R. Wills, IL-10-Secreting CD8+ T Cells Specific for Human Cytomegalovirus (HCMV): Generation, Maintenance and Phenotype. *Pathogens* **11**, 1530

(2022).

54. S. E. Jackson, G. X. Sedikides, G. M. Mason, G. Okecha, M. R. Wills, Human Cytomegalovirus (HCMV)-Specific CD4+ T Cells Are Polyfunctional and Can Respond to HCMV-Infected Dendritic Cells In Vitro. *J. Virol.* **91**, e02128-16 (2017).
